# Structural characterisation and pH-dependent preference of pyrrole cross-link isoforms from reactions of oxoenal with cysteine and lysine side chains as model systems

**DOI:** 10.1007/s00726-023-03295-0

**Published:** 2023-07-11

**Authors:** Malwina Muńko, Karolina Ciesielska, Marcin Hoffmann, Donata Pluskota-Karwatka

**Affiliations:** 1 Center for Advanced Technology, Adam Mickiewicz University, Uniwersytetu Poznańskiego 10, 61-614 Poznan, Poland; 2 Faculty of Chemistry, Adam Mickiewicz University, Uniwersytetu Poznańskiego 8, 61-614 Poznan, Poland

**Keywords:** Pyrrole cross-links, Amino acids, Cysteine, Lysine, Bifunctional carbonyls, 2D NMR

## Abstract

In this study, we subjected 5,5-diethoxy-4-oxopent-2-enal (DOPE), a model amino acids cross-linking reagent, to reactions with *N*-acetylcysteine (Ac-Cys) and *N*^α^-acetyllysine (Ac-Lys), and identified three pyrrole cross-links. The compounds were isolated and their structures were rigorously determined by spectrometric and spectroscopic methods, including 2D NMR experiments. The use of 2D NMR spectroscopy was crucial to determine the position of the substituents in the pyrrole rings. The products were identified as 2,4-, 2,3-, and 2,5-substituted pyrroles. The data obtained from their structural characterisation can help similar studies on amino acids modifications induced by analogous bifunctional carbonyl compounds. Our results show that the study of pathways in which model electrophiles modify amino acids may be helpful for similar studies dealing with identification of structural changes in cysteine- and lysine-containing proteins associated with oxidative stress.

**Supplementary Information:**

The online version contains supplementary material available at 10.1007/s00726-023-03295-0.

## Introduction

Pyrrole cross-links can be formed endogenously by modification of proteins by bifunctional carbonyl compounds such as 4-oxo-2-nonenal and *cis*-2-butene-1,4-dial (Lu and Peterson 2010; Phillips et al. 2014; Zhu et al. 2009). These compounds, derived from different cellular processes, are strong electrophiles capable of interacting with the nucleophilic side chains of proteins, causing cross-linking and/or leading to the formation of covalent adducts (Chen et al. 1997; Zhang et al. 2003). Protein adducts containing a pyrrole moiety are also formed by 4-hydroxy-2-nonenal (Uchida 2003) and 2,5-hexanedione (Pei et al. 2007). The presence of both types of modifications, covalent adducts as well as cross-links, can interfere with the proper functioning of proteins and, consequently, other biomolecules (Nunes et al. 2016; Tabolacci et al. 2012).

**Scheme 1:**
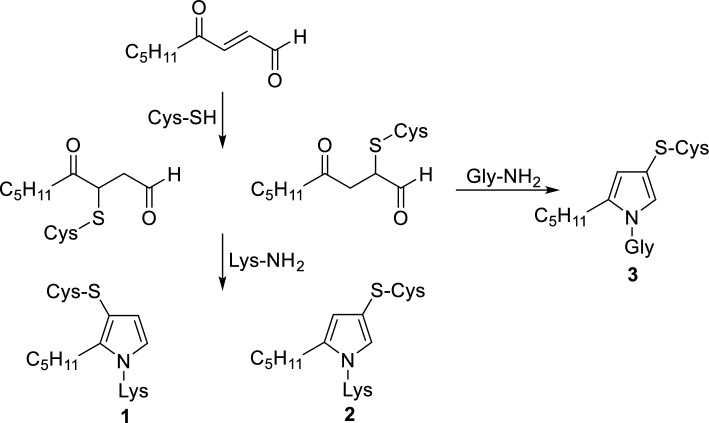
Pyrrole cross-links formed by condensation of 4-ketoaldehyde Michael adducts with lysine (**1** and **2**) or glycine (**3**)

4-Oxo-2-nonenal is one of the most reactive aldehydic products of lipid peroxidation (Lee and Blair 2000). This compound readily undergoes Michael addition to the protein thiol groups of cysteine, giving rise to 4-ketoaldehydes, which in subsequent reactions with biological amines, preferentially the lysine side chains, form substituted pyrroles (Zhang et al. 2003), (pyrrole **1** and **2**, Scheme 1).

Recently, chemometric analysis provided interesting data on the modification of proteins by 4-oxo-2-nonenal and glycine (Sun et al. 2017). The method revealed the formation of a novel pyrrole cross-link to cysteine. The cross-link was identified as a product of Paal Knorr condensation of “4-ketoaldehyde Michael adduct formed by adduction of 4-oxo-2-nonenal to protein cysteinyl thiol, with the amino group of glycine” (Sun et al. 2017), (pyrrole **3**, Scheme 1). This adduct was shown to be the major in vitro product of the 4-oxo-2-nonenal reaction with the proteome (Sun et al. 2017).

Pyrrole cross-links structurally related to those in which formation 4-oxo-2-nonenal is involved are also formed by *cis*-2-butene-1,4-dial, a product of furan bioactivation. Similarly to 4-oxo-2-nonenal, the furan metabolite undergoes initially 1,4-addition of biological thiols forming intermediate adducts, which then condense with the amino groups of proteins yielding 1,3-substituted pyrroles (Lu and Peterson 2010; Peterson et al. 2011). Recently we showed that *cis*-2-butene-1,4-dial undergoes not only a major 1,4-, but also a minor 1,2-addition of a cysteine thiol group. Condensation of the minor adduct with the lysine derivatives yielded 1,2-substituted pyrroles (Muńko et al. 2022). On the basis of their UV spectra and data obtained from LC–MS as well as LC–MS/MS analyses, we tentatively proposed structures of these newly identified cross-links, and we undertook studies aimed at their NMR characterisation. However, as the 1,2-substituted pyrrole cross-links are minor reaction products, their structural characterisation was very challenging. We, therefore, focussed on 5,5-diethoxy-4-oxopent-2-enal (DOPE), a model reagent whose previous use in preliminary studies allowed us to identify this new type of pyrrole cross-links. DOPE is a structural analogue of *cis*-2-butene-1,4-dial, and we expected its reactions with cysteine and lysine derivatives to yield a similar set of pyrrole products. We assumed that the analysis of the NMR spectra of the products will be helpful for future structural studies of analogous products formed in reactions with the furan metabolite. In light of these assumptions, to help elucidate the structure of amino acids modified by *cis*-2-butene-1,4-dial and perhaps other similar bifunctional carbonyl compounds of both endogenous and exogenous origin, this paper describes the structural identification of model pyrroles formed in the reactions of DOPE with cysteine and lysine derivatives.

## Results and discussion

### Preparation of 5,5-diethoxy-4-oxopent-2-enal (DOPE)

5,5-Diethoxy-4-oxopent-2-enal was obtained by treatment of 2-furaldehyde diethyl acetal with 1.5 equivalent of dimethyldioxirane-*d*_6_ (DMDO) in acetone at room temperature using the modified literature procedure (Adger et al. 1991; Druckova and Marnett 2006). For the details, see SI page 22.

### Reaction of DOPE with ***N***-acetylcysteine and ***N***^α^-acetyllysine

**Fig. 1:**
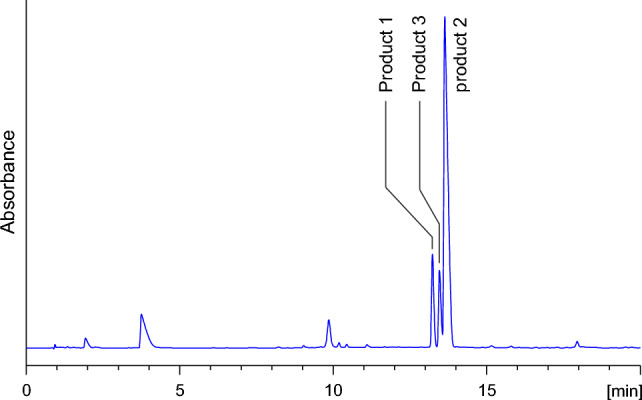
C18 analytical column HPLC chromatogram (recorded at *λ* = 254 nm) of the reaction mixture of DOPE with Ac-Cys and Ac-Lys held in 1 M K_2_HPO_4_/KH_2_PO_4_ (pH 7.4) at 37 °C for 8 h

DOPE was subjected to the reaction with equal molar amounts of *N*-acetylcysteine (Ac-Cys) and *N*^α^-acetyllysine (Ac-Lys) in 1 M K_2_HPO_4_/KH_2_PO_4_ at pH 7.4 and 37 °C. Reverse-phase HPLC analysis of the reaction mixture revealed the presence of three major products named product 1, product 2, and product 3 (Fig. 1), which achieved maximum concentration after 16 h.

The UV spectra of the three compounds were different (SI, Fig. S26 and S27), whilst in their mass spectra (negative mode), signals assigned to the [M–H]^–^ ions were observed at *m/z* = 426.1343 for product 1 and product 2, and at *m/z* = 426.1342 for product 3 (ESI, Figs. S1, S2, and S3, respectively). The measured m/z values corresponded to the same molecular formula of C_18_H_25_N_3_O_7_S for all three compounds. (Mass spectra recorded in the positive ion mode (SI, Figs. S28–30) led to the same conclusion). However, in view of the literature data, cross-links structurally similar to the pyrroles shown in Scheme 1 were expected to form. Such compounds should have the molecular formula of C_22_H_36_N_3_O_8_S and generate an [M-H]^–^ signal at *m*/*z* = 500.2067 (calculated value). The difference between the molecular patterns of four carbon atoms, ten hydrogen atoms, and one oxygen atom suggested the absence of an acetal moiety in the resulting compounds. Based on these observations, we assumed that the products contain an aldehyde HC=O group instead of HC(OCH_2_CH_3_)_2_. The three compounds also had a similar fragmentation pattern (SI, Figs. S1, S2, and S3). The most intensive were the signals observed at *m/z* = 297.0911 for product 1 and product 2, and at *m/z* = 297.0918 for product 3, which were consistent with cleavage of the bonds between the sulphur atoms and the methylene group carbon atoms (*β*) in the cysteine residues. The MS/MS spectrum of product 1 also showed the signals at *m/z* = 269.0959 and 227.0848. The first one corresponded to the loss of the aldehyde group from the product ion whose signal appeared at *m/z* = 297.0911. The product ion *m*/*z* = 227.0848 corresponded to the loss of the acetyl group from the product ion at *m*/*z* = 269.0959 (SI Fig. S1). The signals observed in the MS/MS spectra of product 2 and product 3 at *m/z* = 255.0798 and 255.0802, respectively, were attributed to the loss product of acetyl from the product ions at *m/z* = 297.0911 and 297.0918. Based on the similarity of the MS/MS spectra of all three main reaction products, we assumed that the identified compounds were structural analogues.

To confirm the hypothesis of structural similarity and explore the subtle differences between their structures, all compounds were subjected to NMR studies (the NMR spectra obtained for the compounds are presented in SI Figs. S7–S21).

Analysis of the ^1^H NMR spectra of product 1 and product 2 showed the presence of signals derived from substituted pyrrole rings. In the spectrum of the first compound, signals attributed to this ring were observed at *δ* = 6.43 ppm and *δ* = 7.34 ppm (SI Fig. S7), whilst in the spectrum of the second product, they were seen at *δ* = 7.21 ppm and *δ* = 7.34 ppm (SI Fig. S12). Moreover, in the ^1^H NMR spectra of both compounds, no signals of acetal groups were found. Instead, signals appearing at about *δ* = 9 ppm, assigned to aldehyde protons, were present (SI Figs. S7, S12).

The literature data suggest that the values of the coupling constants between protons in the pyrrole ring can determine the positions of substituents (Chadwick 2008). Values in the range of 1.35–1.80 Hz can indicate the presence of substituents at positions C2 and C4, whilst in the case of substituents present at C2 and C3, these values are in the range of 2.40–3.10 Hz. The highest values (3.40–3.80 Hz) are observed for the pyrrole ring substituted at C2 and C5 (Chadwick 2008).

**Fig. 2:**
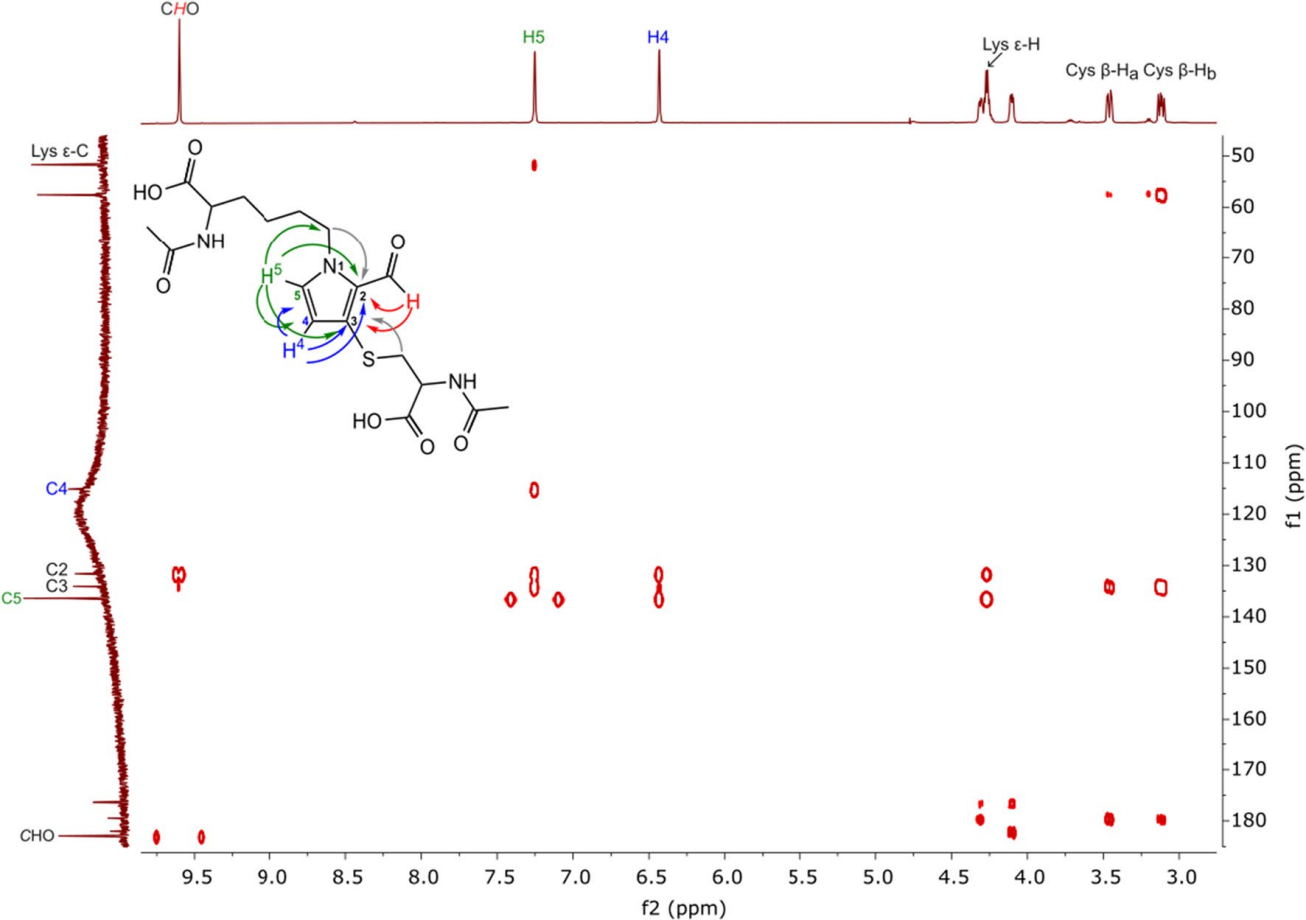
Major correlations observed in the HMBC spectrum (D_2_O) of product 1

In the ^1^H NMR spectrum of product 1, signals derived from the pyrrole ring protons were observed as doublets with the coupling constant equal to 1.80 Hz, whilst in the spectrum of product 2, one proton signal was seen as a doublet with the coupling constant equal to 1.72 Hz; however, a signal derived from the second proton was observed as a broad singlet making it impossible to determine the value of the coupling constant. Coupling constants equal to 1.80 Hz and 1.72 Hz suggested the substitution of C2 and C4 positions in both compounds. Consequently, the method based on the values of the coupling constants was ineffective in determining the position of the substituents in the pyrrole ring of the compounds under study. Methods based on 2D NMR spectroscopy were, therefore, used. The signal observed in the ^13^C NMR spectrum of product 1 at *δ* = 131.61 (SI Fig. S8) was assigned to the quaternary carbon atom C2 based on the intensive correlation in the HMBC spectrum between this signal and the signal derived from the aldehyde proton (Fig. 2).

**Fig. 3:**
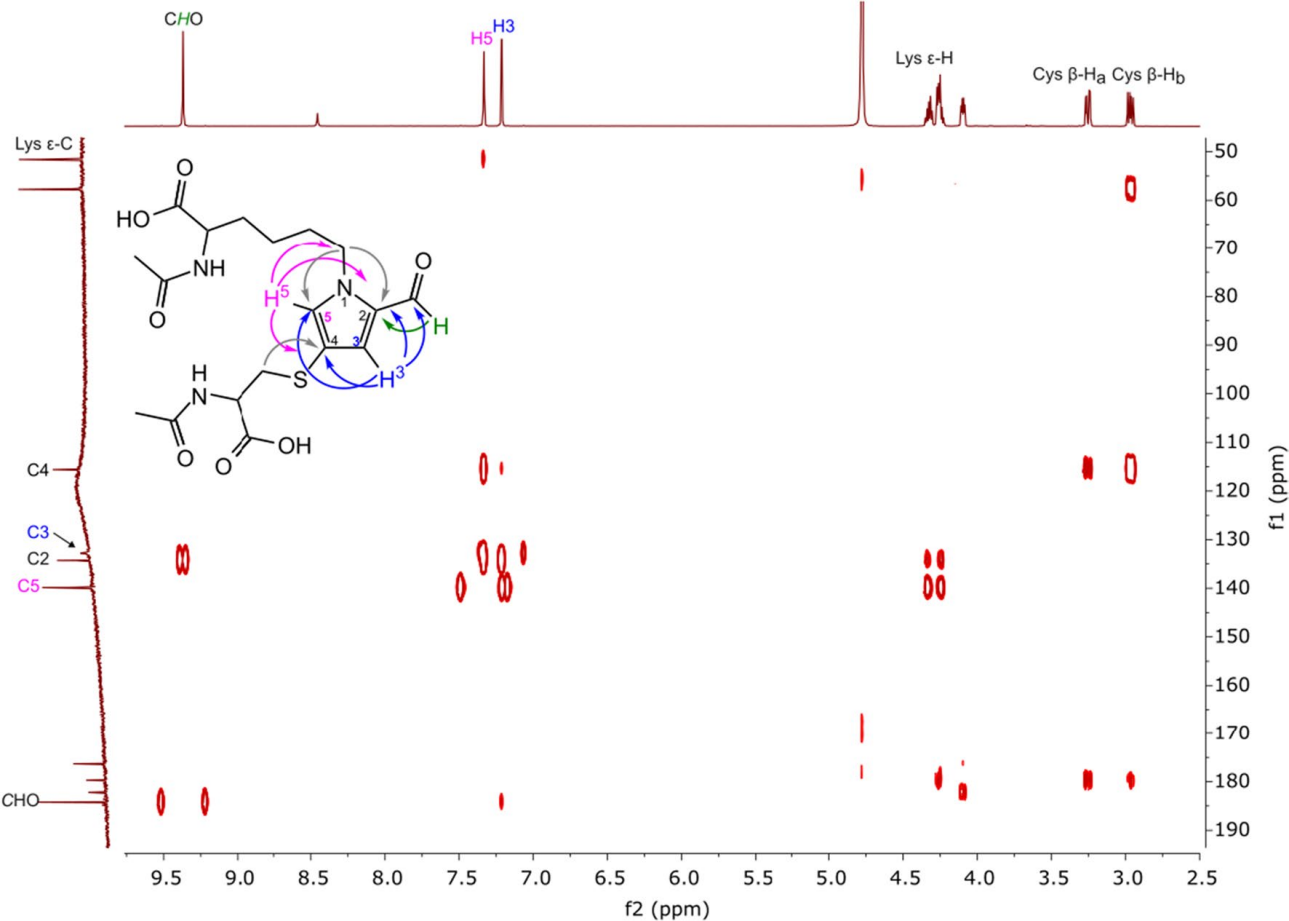
Major correlations observed in the HMBC spectrum (D_2_O) of product 2

In the HMBC spectrum, a weak correlation signal between the signal of the aldehyde proton and the quaternary carbon atom at *δ* = 134.07 ppm was also observed (Fig. 2). The signal was assigned to C3. This signal correlated with the signals derived from the diastereotopic protons of the β-methylene group of *N*-acetylcysteine. This correlation proved the presence of a cysteine substituent on C3. HSQC spectrum of product 1 (SI Fig. S10) revealed correlations between the one proton doublet at *δ* = 7.26 ppm and the signal of carbon atom appeared at *δ* = 136.33 ppm, as well as between the one proton doublet at *δ* = 6.43 ppm and the signal of a carbon atom at *δ* = 115.12 ppm. Analysis of the HMBC spectrum showed that the two protons are 2–3 chemical bonds away from the C2 and C3 carbon atoms but did not allow these protons to be assigned the value of chemical shifts. The key correlation was observed between the proton signal at *δ* = 7.26 ppm and the signal of the carbon atom of the ε-methylene group of *N*^α^-acetyllysine. The analogous correlation between this carbon atom signal and the proton signal at *δ* = 6.43 ppm was not observed. Based on these findings, the signal at *δ* = 7.26 ppm was assigned to H-5, and the signal at *δ* = 6.43 ppm was assigned to H-4. This assignment was confirmed by the correlation signal observed between these two protons in the COSY spectrum (SI Fig. S9). Using the same approach, the positions of substituents in product 2 were determined. The structure and major correlations observed in the HMBC spectrum of product 2 are presented in Fig. 3.

**Fig. 4:**
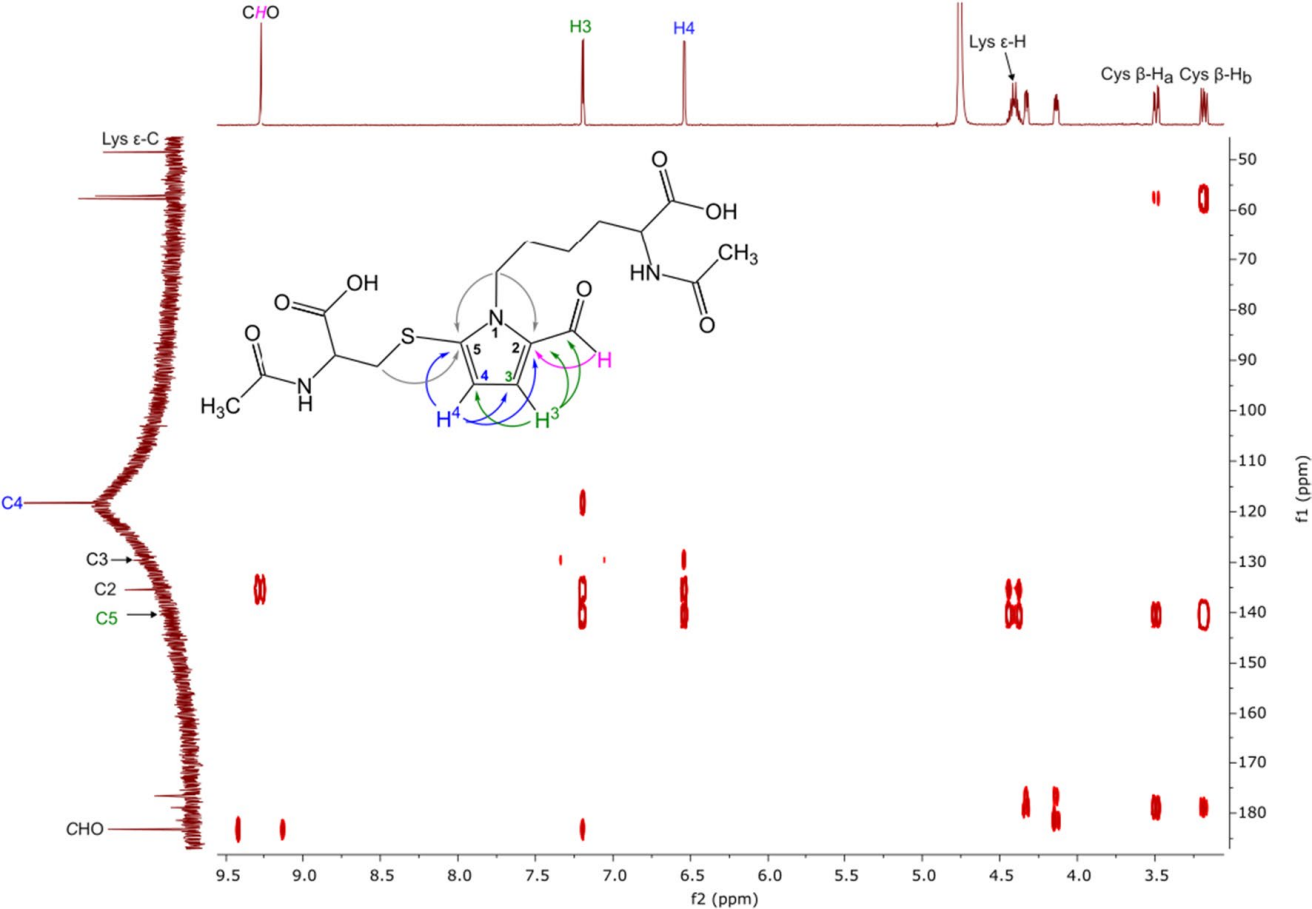
Major correlations observed in the HMBC spectrum (D_2_O) of product 3

One-proton singlet observed in the ^1^H NMR spectrum of product 3 at *δ* = 9.28 ppm (SI Fig. S17) was assigned to the aldehyde proton based on the chemical shift and one-bond correlation with the carbon atom whose signal appeared in the ^13^C NMR spectrum at *δ* = 183.21 ppm (SI Fig. S18 and S20). Two doublets observed at *δ* = 6.54 and *δ* = 7.19 ppm were assigned to the protons of the pyrrole ring. The signals showed a relatively high coupling constant (*J* = 4.28 Hz), indicating the presence of substituents at the 2 and 5 positions. The assignment was confirmed by the presence in the COSY spectrum of the correlation signals observed for these two protons (SI, Fig. S19). In the HMBC spectrum, the correlation between the proton signal at *δ* = 6.54 ppm and the signals of C2, C3, and C5, respectively, were observed (SI Fig. S21). However, no correlation was observed between this proton signal and the aldehyde carbon atom signal. On the basis of these findings, the proton signal at *δ* = 6.54 ppm was assigned to H-C4 (Fig. 4), whilst the proton signal at *δ* = 7.19 ppm, which showed a correlation with the aldehyde carbon atom signal, was attributed to H-C3.

### Formation of the pyrrole cross-links

**Scheme 2:**
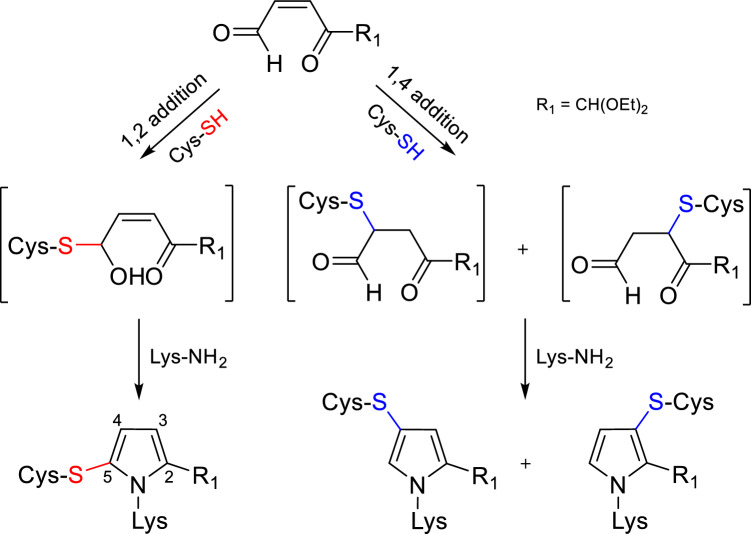
Routes proposed for the formation of pyrrole cross-links by the model oxoenal with* N*-acetylcysteine and *N*^α^-acetyllysine

Formation of product 1, product 2, and product 3 was most likely initiated, respectively, by the Michael type and 1,2-addition of the *N*-acetylcysteine thiol group to the oxoenal used. The building of the pyrrole ring was then accomplished by the reaction of *N*^α^-acetyllysine with the addition product formed (Scheme 2).

**Scheme 3:**
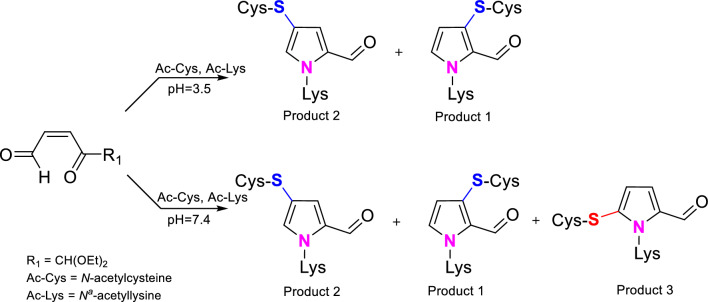
pH-dependent formation of product 1, product 2, and product 3

Our studies showed that the course of the cross-links formation is affected by pH of the reaction medium, and that the pH determines the regioselectivity of the thiol group addition. Under acidic conditions (pH 3.5), 1,4-addition took place leading to the formation of product 1 and product 2. At pH 7.4, product 3 arising from 1,2 addition was also formed beside the cross-links resulted from the 1,4-addition (Scheme 3).

**Fig. 5:**
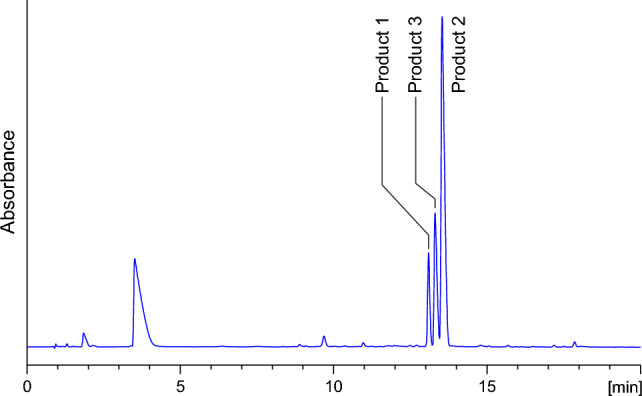
C18 analytical column HPLC chromatogram (recorded at *λ* = 254 nm) of the reaction mixture of DOPE with *N*-acetylcysteine and *N*^α^-acetyllysine held at pH 10 and 37 °C for 8 h

An explanation of such regioselectivity seems to be obvious; at pH 3.5, thiol group, belonging to so-called soft nucleophiles, prefers to react with a softer electrophilic centre, the β-carbon atom in the α,β-unsaturated system. At pH 7.4, this group can be partially deprotonated; therefore, the probability of its acting as a harder nucleophile, that chooses to react with the carbonyl carbon atom (harder electrophilic centre) yielding product 3, increases. Support for such an explanation is provided by the fact that under basic conditions (pH 10), which favour the thiol group deprotonation, marked increase in the formation of the 1,2-addition product was observed (Fig. 5). Based on the area of the corresponding signals in the chromatogram shown in Fig. 5, the approximate ratio of products 1, 2, and 3 can be estimated as 2:10:3. pH also affects the reactivity of the Ac-Lys amino group. Under acidic conditions, the group undergoes partial protonation and loses some of its nucleophilic character. At neutral pH, its reactivity is sufficient to trap both 1,4- and 1,2-addition products.

**Scheme 4:**
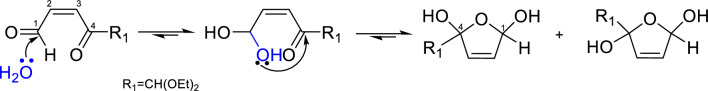
Proposed mechanism for the formation of the DOPE cyclic hydrates

Independently on the reaction pH, product 2, 2,4-substituted pyrrole, was the major product. It should not be surprising due to the fact that the cross-link is formed by addition to the less hindered β-carbon atom. On the other hand, in model studies on protein side chain modification induced by 4-oxo-2-nonenal, 2,3-substituted pyrroles were found to be the major cross-links formed (Zhang et al. 2003). This shows that apart from the steric hindrance, another factor contributes to the regioselectivity of the reactions with DOPE. The factor can be associated with hydration to which DOPE is prone. Our studies show that at pH 7.4, DOPE readily undergoes hydration forming cyclic products (Scheme 4, for the details see SI page 23). The plausible mechanism implies the addition of water molecule to the carbonyl carbon atom in the DOPE aldehyde group followed by an intramolecular attack of the attached hydroxyl group at the carbonyl carbon atom of the keto function. This leads to the ring closure and cyclic hydrates formation (Scheme 4).

Due to involvement of the DOPE aldehyde group into this process, Michael type addition is preferred to C2. It seems that this preference influences regioselectivity of the pyrrole cross-links formation stronger than the factor associated with the steric hindrance. This is in agreement with the finding that unlike DOPE, 4-oxo-2-nonenal was reported to exist in the reaction medium predominantly in the aldehyde form. Therefore, in this compound, due to the greater electrophilicity of the enal functionality compared to the enone one, C3 exhibits higher reactivity in conjugate addition leading to the formation of 2,3-substituted pyrroles as the main cross-linked products (Zhang et al. 2003). All these results indicate a significant influence of the bifunctional electrophile structure on the course of the cross-links formation.

An interesting issue associated with the DOPE cross-links formation is the presence in their molecules of aldehyde group instead of the expected acetal function. A plausible explanation is provided by the studies showing that unlike cyclic acetals, a variety of dimethyl and diethyl acetals, including aromatic ones, undergo quantitative hydrolysis in aqueous solutions at ambient temperature without a catalyst or any other additive (Williams et al. 2010). It is most likely that such a process also occurs with DOPE during its reactions with amino acids.

## Conclusion

In this study, the reactivity of 5,5-diethoxy-4-oxopent-2-enal (DOPE), a model cross-linking reagent, towards the formation of pyrrole cross-links with *N*-acetylcysteine (Ac-Cys) and *N*^α^-acetyllysine (Ac-Lys) was investigated and structures of the products identified were strictly determined by spectroscopic and spectrometric methods. As expected, DOPE underwent Michael-type addition at the C-2 and C-3 positions and 1,2-addition to the aldehyde group, yielding two 1,4- and one 1,2-addition products when reacting with Ac-Cys. Upon condensation with the amine group of Ac-Lys, the addition products gave 2,4-, 2,3- and 2,5-substituted pyrroles, respectively. Determining the position of the substituents was non-trivial and only possible on the basis of careful analysis of data obtained from 2D NMR experiments.

Model systems used in scientific research are not always perfect, but they provide insight into similar events that may occur with biologically relevant molecules. Thus, the use of DOPE as a model amino acid cross-linking reagent in our earlier studies has shed new light on the reactivity of the furan metabolite (Muńko et al. 2022). In the present work, this approach provides general, important data on the structural and mechanistic aspects of pyrrole cross-link formation. These data can not only help in the structural characterisation of the recently identified cross-link formed by *cis*-2-buten-1,4-dial, but also provide valuable support in the chemistry and structure determination of various differently substituted pyrrole derivatives.

## Experimental

### Chemicals

Dimethyldioxirane (DMDO) was prepared as previously reported (Adger et al. 1991; Druckova and Marnett 2006). Briefly, peroxymonosulphate (Oxone, 50 g, 0.08 mol) was added in about 15 min in three portions to a stirred mixture of water (40 mL), acetone (24 mL), and sodium bicarbonate (24 g) under an argon atmosphere. The reaction proceeded for 5 min, and vacuum was applied to the reaction assembly. The slightly yellow-coloured distillate was collected over 1 h in the receiving flask and cooled in a dry ice/i-PrOH bath. The solution was dried over MgSO_4_ and stored at − 20 °C for no more than 24 h prior to use. DMDO content was assayed by the use of iodometric titration, usually affording concentrations of 0.05 M. Acetone-*d*_6_ was used to prepare deuterated DMDO.

All other reagents were purchased from Sigma-Aldrich.

Demineralized water was used to prepare the buffer solutions. The water was filtered under reduced pressure through a Nylon 66 Filter Membrane (Supelco Analytical).

### Chromatographic methods

Progress of the reactions was monitored by HPLC. The analyses were performed on an Agilent 1100 series liquid chromatographic system consisting of a binary pump (G1312A), autosampler (G1313A), vacuum degasser (G1379A), diode-array detector (UV; G1315B), a 5 μm, 4.6 × 150 mm reversed-phase C18 anal. column (Hypersil BDS, Thermo Scientific) and Agilent ChemStation data handling programme (Agilent Technologies). The column was eluted isocratically for 5 min with 0.01 M phosphate buffer, pH 7 (solvent A), and then with a gradient from 0 to 30% MeCN (solvent B) in 30 min at a flow rate of 1.5 ml/min.

Separation and purification of the synthesised compounds were carried out using an Agilent 1200 Series HPLC system consisting of a binary pump (G1312A), vacuum degasser (G1379B), autosampler (G1329A), thermostated column compartment (G 1316A), diode-array detector (UV; G1315B), fraction collector (G1364C), and Agilent ChemStation data handling programme (Agilent Technologies). 5 mM NH_4_HCO_3_ (solvent A) and MeCN (solvent B) were used as eluents. The flow was set to 4 mL/min. Isolation of product 1 and product 2 was performed using a semiprep. 5 μm, 10 × 250 mm (Hypersil GOLD, Thermo Scientific) reversed-phase C18 column. Isolation of product 3 was carried out by the use of a semiprep. 5 μm, 10 × 250 mm (Hypersil BDS, Thermo Scientific) reversed-phase C18 column.

### ESI-LC/MS and ESI-LC/MS/MS analyses

The ESI-LC/MS and ESI-LC/MS/MS analyses were performed with Agilent 1200 LC system with Agilent Q-TOF 6540 spectrometer with DUAL AJS ESI source. Ionisation was carried out using nitrogen as nebulizing/drying gas (8 L/min, 300 °C, 35 psi) and sheath gas (11 L/min, 325 °C). The VCap was set to 3500 V, the fragmentor was set to 100 V. Ion polarity mode and collision energy values for MS/MS experiments were chosen individually and given with the appropriate spectra. The analysed compounds were introduced through the LC system using a reversed-phase analytical column (5 μm, 4.6 mm × 150 mm, Hypersil GOLD, Thermo Scientific) eluted isocratically for 2 min with 1.2 mM CH_3_COONH_4_ (solvent A) and then with a gradient from 0 to 30% of MeCN (solvent B) for 25 min with a flow rate of 0.5 min/min. The thermostat was set to 25 °C.

### NMR spectroscopy

Samples of compounds subjected to NMR studies were dissolved in D_2_O (internal standard: 3-(trimethylsilyl)propionic-2,2,3,3-*d*_4_ acid sodium salt, TSP-*d*_4_), 0.1 M K_2_DPO_4_/KD_2_PO_4_ in D_2_O at pH^*^ 7.4 (internal standard: TSP-*d*_4_), or in (CD_3_)_2_CO (internal standard: 0.03% TMS). The experiments were performed at the temperature of 298 K. All spectra were acquired on a Bruker Avance DRX 600 system, operating at frequencies of 600.3 MHz (^1^H) and 150.9 MHz (^13^C). The spectrometer was equipped with 5 mm triple-resonance inverse probe head [^1^H/^31^P/BB]. High-power ^1^H, ^13^C π/2 pulses of 9.00 and 15.00 μs, respectively, were used. 1D and 2D homonuclear and heteronuclear correlation experiments were carried out using pulse sequences from the Bruker pulse programme library (COSY pulse programme: cosygpqf; HSQC pulse programme: hsqcetgp; HMBC pulse programme: hmbcgplpndqf). COSY spectra were acquired using the following parameters: time domain points 2048, number of transients per increment 64, 256 increments in t1. In HSQC spectra, 1024 data points along direct dimension (*F*_2_) and 256 increments along indirect dimension (*F*_1_) were acquired with 96 transients per increment. In HMBC spectra, 4096 data points with 128 scans per increment and 256 increments were acquired, and the D1 delay was set to 3.45 ms.

### General procedure for determination of DMDO concentration by iodometric titration

1 mL of the obtained DMDO (solution in acetone) was added to 2 mL of a mixture of glacial acetic acid with acetone (3:2 (v:v)), next 2 mL of saturated water solution of KI was introduced. The resulted mixture was held in the dark at room temperature for 10 min. Then 5 mL of water was added. 1 mL of the mixture was taken out and subjected to titration with Na_2_S_2_O_3_ (0.001 N) until the yellow colour disappeared. Titration was performed three times. On the basis of the obtained results, concentration of DMDO was calculated. Usually, the concentration was equal to 0.05–0.08 M.

### General procedure for synthesis of 5,5-diethoxy-4-oxopent-2-enal (DOPE)

2-Furaldehyde diethyl acetal (85 mg, 0.5 mmol) was dissolved in 2 mL of acetone under an argon atmosphere, and 10 mL (37 mg, 0.5 mmol) of DMDO was added at once. The reaction proceeded at room temperature typically for 2 h. After that time, the total volume was reduced to 3 mL. When the synthesised compound was subjected to NMR analysis, acetone-*d*_6_ was used as a solvent for 2-furaldehyde diethyl derivative as well as a reagent for the preparation of DMDO.

^1^H NMR (600 MHz; acetone-*d*_6_): δ: 10.14 (1 H, d, 1-H, *J*_1,2_ = 7.1 Hz), 7.35 (1 H, d, 3-H, *J*_3,2_ = 12.0 Hz), 6.36 (1 H, dd, 2-H, *J*_2,1_ = 7.1 Hz, *J*_2,3_ = 12.0 Hz), 4.86 (1 H, s, 5-H), 3.77 (2 H, dq, a-H, *J* = 7.1 Hz and 9.5 Hz), 3.35 (2 H, dq, b-H, *J* = 7.1 Hz and 9.5 Hz), 1.21 (3 H, s, Me).

### Synthesis of product 1 and product 2

*N*-Acetylcysteine (98 mg, 0.6 mmol) and *N*^*α*^-acetyllysine (113 mg, 0.6 mmol) were dissolved in 1 M phosphate buffer solution (6 mL, pH 7.4) and heated to 37 °C. Then 3 mL of an acetone solution of DOPE (75 mg, 0.4 mmol) was added. Acetone was removed in a stream of argon and the resulted reaction mixture was heated for 11 h. The cross-linking products were isolated and purified by the use of HPLC. The HPLC column was eluted with a gradient from 0 to 20% of MeCN for 22 min. The fractions containing pure products were respectively combined, evaporated to dryness, and lyophilized. The resulting product 1 (4.0 mg, yield: 2.3%) and product 2 (13.8 mg, yield: 8.1%) were isolated as light orange solids and subjected to spectroscopic and spectrometric studies.

*Product 1* ^1^H NMR (600 MHz, D_2_O) δ 9.60 (s, 1H, C*H*O), 7.25 (d, *J* = 1.7 Hz, 1H, C5-H), 6.43 (d, *J* = 1.9 Hz, 1H, C4-H), 4.31 (dd, *J* = 8.8, 3.5 Hz, 1H, Cys α-C*H*), 4.26 (m, 3H, Lys ε-C*H*_*2*_), 4.10 (dd, *J* = 9.0, 4.4 Hz, 1H, Lys α-C*H*), 3.46 (dd, *J* = 14.1, 3.6 Hz, 1H, Cys β-C*H*_*a*_), 3.12 (dd, *J* = 14.0, 9.0 Hz, 1H, Cys β-C*H*_*b*_), 1.98 (s, 3H, Lys C*H*_*3*_CO), 1.90 (s, 3H, Cys C*H*_*3*_CO), 1.82–1.69 (m, 3H, Lys δ-C*H*_2_, Lys β-C*H*_*a*_), 1.63 (m, 1H, Lys β-C*H*_*b*_), 1.31–1.19 (m, 2H, Lys γ-C*H*_2_). HRMS calc. for C_18_H_24_N_3_O_7_S^–^ [M–H]^–^ *m/z* = 426.1335, found *m/z* = 426.1343 (error 1.88 ppm). UV: *λ*_max_ = 298 nm, *λ*_min_ = 247 nm.

*Product 2* ^1^H NMR (600 MHz, D_2_O) δ 9.37 (s, 1H, C*H*O), 7.33 (s, 1H, C5-H), 7.21 (d, *J* = 1.7 Hz, 1H), 4.33 (m, 1H, Lys ε-C*H*_*a*_), 4.28–4.21 (m, 2H, Cys α-C*H*, Lys ε-C*H*_*b*_), 4.10 (dd, *J* = 9.3, 4.4 Hz, 1H, Lys α-C*H*), 3.25 (dd, *J* = 14.0, 3.8 Hz, 1H, Cys β-C*H*_*a*_), 2.97 (dd, *J* = 14.0, 8.8 Hz, 1H, Cys β-C*H*_*b*_), 1.99 (s, 3H, Cys C*H*_*3*_CO), 1.95 (d, *J* = 3.8 Hz, 3H, Lys C*H*_*3*_CO), 1.82–1.70 (m, 3H, Lys β-C*H*_*a*_*,* Lys γ-C*H*_2,_), 1.63 (m, 1H, Lys β-C*H*_*b*_), 1.28–1.24 (m, 2H, Lys δ-C*H*_2_). HRMS calc. for C_18_H_24_N_3_O_7_S^–^ [M–H]^–^ *m/z* = 426.1335, found *m/z* = 426.1343 (error 1.88 ppm). UV: *λ*_max_ = 265 and 301 nm, *λ*_min_ = 229 and 285 nm.

### Synthesis of product 3

*N*-Acetylcysteine (86 mg, 0.53 mmol) and *N*^*α*^-acetyllysine (100 mg, 0.53 mmol) were dissolved in 1 M phosphate buffer solution (6 mL, pH 7.4). Then 3 mL of an acetone solution of DOPE (65 mg, 0.35 mmol) was added. The reaction mixture was incubated at 37 °C for 16 h. DOPE-NAC-NAAL-3 was isolated and purified by the use of HPLC. The HPLC column (thermostated at 25 °C) was eluted with a gradient from 0 to 12% of ACN for 22 min and then from 12 to 20% of MeCN for 3 min at a flow rate of 4 mL/min. The isolated product was subjected to further purification. The HPLC column (thermostated at 20 °C) was eluted isocratically with 4% of MeCN for 15 min at a flow rate of 2.5 mL/min. The fractions containing pure product were combined, evaporated to dryness, and lyophilized. Product 3 was obtained in the form of a light orange solid (4.5 mg, yield: 2.8%).

^1^H NMR (600 MHz, D_2_O) δ 9.28 (s, 1H, C*H*O), 7.19 (d, *J* = 4.3 Hz, 1H, C3-H), 6.54 (d, *J* = 4.3 Hz, 1H, C4-H), 4.41 (m, 2H, Lys ε-C*H*_*2*_), 4.33 (dd, *J* = 8.5, 3.8 Hz, 1H, Cys α-C*H*), 4.14 (dd, *J* = 9.4, 4.6 Hz, 1H, Lys α-C*H*), 3.49 (dd, *J* = 14.2, 3.9 Hz, 1H, Cys β-C*H*_*a*_), 3.19 (dd, *J* = 14.2, 8.6 Hz, 1H, Cys β-C*H*_*b*_), 1.98 (s, 3H, Lys C*H*_*3*_CO), 1.90 (s, 3H, Cys C*H*_*3*_CO), 1.82–1.75 (m, 1H, Lys β-C*H*_*a*_), 1.71–1.60 (m, 3H, Lys δ-C*H*_2_, Lys β-C*H*_*b*_), 1.28–1.19 (m, 2H, Lys γ-C*H*_2_). HRMS calc. for C_18_H_24_N_3_O_7_S^–^ [M–H]^–^: 426.1335, found *m/z* = 426.1342 (error 1.6 ppm). UV: *λ*_max_ = 320 nm, *λ*_min_ = 236 nm.

### Investigation of the effect of pH on the course of the reactions of DOPE with ***N***‐acetylcysteine and ***N***^***α***^‐acetyllysine (analytical scale)

*N*‐Acetylcysteine (9.8 mg, 0.06 mmol) and *N*^*α*^‐acetyllysine (11.3 mg, 0.06 mmol) were dissolved in 1 M K_2_HPO_4_/KH_2_PO_4_ (1 mL, pH 3.5) and heated to 37 °C. Then 0.5 mL of an acetone solution of DOPE (7.5 mg, 0.04 mmol) was added. Acetone was removed under a stream of argon, the reaction mixture was heated for 16 h. Progress of the reaction was monitored by HPLC and LC–MS.

### Supplementary Information

Below is the link to the electronic supplementary material.

Supplementary file1 (DOCX 1350 kb)

## Data Availability

Electronic supplementary information including MS, UV, and NMR spectra of the compounds obtained is available.

## References

[CR1] Adger BM, Barrett C, Brennan J, McKervey MA, Murray RW (1991). Oxidation of furans with dimethyldioxirane. J Chem Soc Chem Commun.

[CR2] Chadwick D (2008). Physical and theoretical aspect of 1H-pyrroles. In chemistry of heterocyclic compounds.

[CR3] Chen L-J, Hecht SS, Peterson LA (1997). Characterization of amino acid and glutathione adducts of *cis*-2-butene-1,4-dial, a reactive metabolite of furan. Chem Res Toxicol.

[CR4] Druckova A, Marnett LJ (2006). Characterization of the amino acid adducts of the enedial derivative of teucrin A. Chem Res Toxicol.

[CR5] Lee SH, Blair IA (2000). Characterization of 4-oxo-2-nonenal as a novel product of lipid peroxidation. Chem Res Toxicol.

[CR6] Lu D, Peterson LA (2010). Identification of furan metabolites derived from cysteine-*cis*-2-butene-1,4-dial-lysine cross-links. Chem Res Toxicol.

[CR7] Muńko M, Ciesielska K, Pluskota-Karwatka D (2022). New insight into the molecular mechanism of protein cross-linking induced by cis-2-butene-1,4-dial, the metabolite of furan: Formation of 2-substituted pyrrole cross-links involving the cysteine and lysine residues. Bioorg Chem.

[CR8] Nunes J, Martins IL, Charneira C, Pogribny IP, de Conti A, Beland FA, Marques MM, Jacob CC, Antunes AMM (2016). New insights into the molecular mechanisms of chemical carcinogenesis: In vivo adduction of histone H2B by a reactive metabolite of the chemical carcinogen furan. Toxicol Lett.

[CR9] Pei W, Misumi J, Kubota N, Morikawa M, Kimura N (2007). Two new reactive targets of 2,5-hexanedione in vitro-beta-alanine and glycine. Amino Acids.

[CR10] Peterson LA, Phillips MB, Lu D, Sullivan MM (2011). Polyamines are traps for reactive intermediates in furan metabolism. Chem Res Toxicol.

[CR11] Phillips MB, Sullivan MM, Villalta PW, Peterson LA (2014). Covalent modification of cytochrome *c* by reactive metabolites of furan. Chem Res Toxicol.

[CR12] Sun R, Fu L, Liu K, Tian C, Yang Y, Tallman KA, Porter NA, Liebler DC, Yang J (2017). Chemoproteomics reveals chemical diversity and dynamics of 4-oxo-2-nonenal modifications in cells. Mol Cell Proteom.

[CR13] Tabolacci C, Lentini A, Provenzano B, Beninati S (2012). Evidences for a role of protein cross-links in transglutaminase-related disease. Amino Acids.

[CR14] Uchida K (2003). Histidine and lysine as targets of oxidative modification. Amino Acids.

[CR15] Williams DBG, Cullen A, Fourie A, Henning H, Lawton M, Mommsen W, Nangu P, Parker J, Renison A (2010). Mild water-promoted selective deacetalisatison of acyclic acetals. Green Chem.

[CR16] Zhang W-H, Liu J, Xu G, Yuan Q, Sayre LM (2003). Model studies on protein side chain modification by 4-oxo-2-nonenal. Chem Res Toxicol.

[CR17] Zhu X, Gallogly MM, Mieyal JJ, Anderson VE, Sayre LM (2009). Covalent cross-linking of glutathione and carnosine to proteins by 4-oxo-2-nonenal. Chem Res Toxicol.

