# Supplementary material for: Structural characterisation and pH-dependent preference of pyrrole cross-link isoforms from reactions of oxoenal with cysteine and lysine side chains as model systems
Source: Amino Acids. 2023 Jul 11;55(9):1073–82. doi: 10.1007/s00726-023-03295-0 (PMC10564825; doi:10.1007/s00726-023-03295-0)
Supplement: Supplementary file 1 — Supplementary file1 (DOCX 1350 kb) [file 726_2023_3295_MOESM1_ESM.docx]

**supplementary information**

**Structural characterisation and pH-dependent preference of pyrrole
cross-link isoforms from reactions of oxoenal with cysteine and lysine side chains as model systems**

Malwina Muńko ^a^, Karolina Ciesielska ^b^, Marcin Hoffmann ^b^, Donata Pluskota-Karwatka ^b,^*

^a^ Center for Advanced Technology, Adam Mickiewicz University, Uniwersytetu Poznańskiego 10, 61-614 Poznań, Poland

^b^ Adam Mickiewicz University, Faculty of Chemistry, Uniwersytetu
Poznańskiego 8, 61-614 Poznań, Poland

1. MS/MS spectra of the studied cross-links **2**
2. NMR spectra of the studied cross-links **3**
3. Preparation of DOPE **22**
4. Hydration of DOPE **23**
5. UV spectra of the studied compounds **26**
6. Mass spectra of the studied compounds **27**

**Mass spectra of the studied compounds**

**Fig. S1. Negative ions MS/MS spectrum of product 1, collision energy 15 eV.**

The inset in the figure shows the structure proposed for product 1 based on knowledge of the reactivity of the conjugated unsaturated carbonyl compounds with nucleophilic molecules in combination with data obtained from the MS/MS spectrum shown above.

**Fig. S2. Negative ions MS/MS spectrum of product 2, collision energy 15 eV**

The inset in the figure shows the structure proposed for product 2 based on knowledge of the reactivity of the conjugated unsaturated carbonyl compounds with nucleophilic molecules in combination with data obtained from the MS/MS spectrum shown above.

**Fig. S3. Negative ions MS/MS spectrum of product 3; collision energy 15 eV**

The inset in the figure shows the structure proposed for product 3 based on knowledge of the reactivity of the conjugated unsaturated carbonyl compounds with nucleophilic molecules in combination with data obtained from the MS/MS spectrum shown above.

**NMR spectra**

**
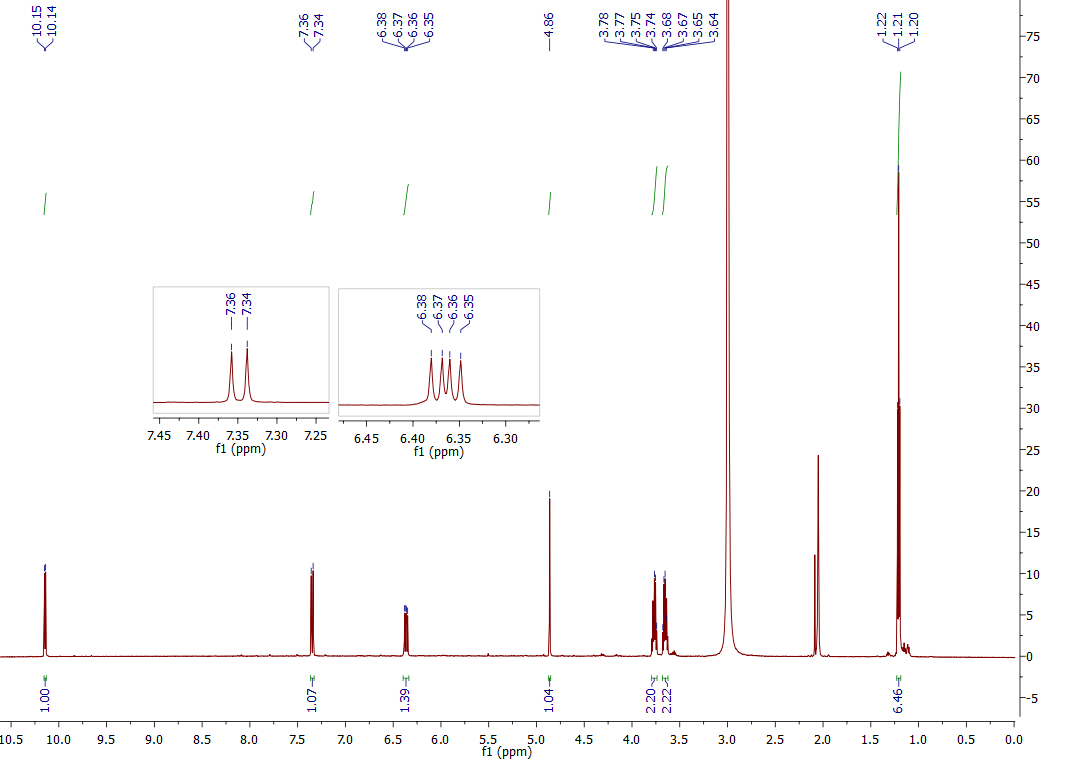
**

**Fig S4. ^1^H NMR spectrum of DOPE (acetone-*d_6_*)**

**
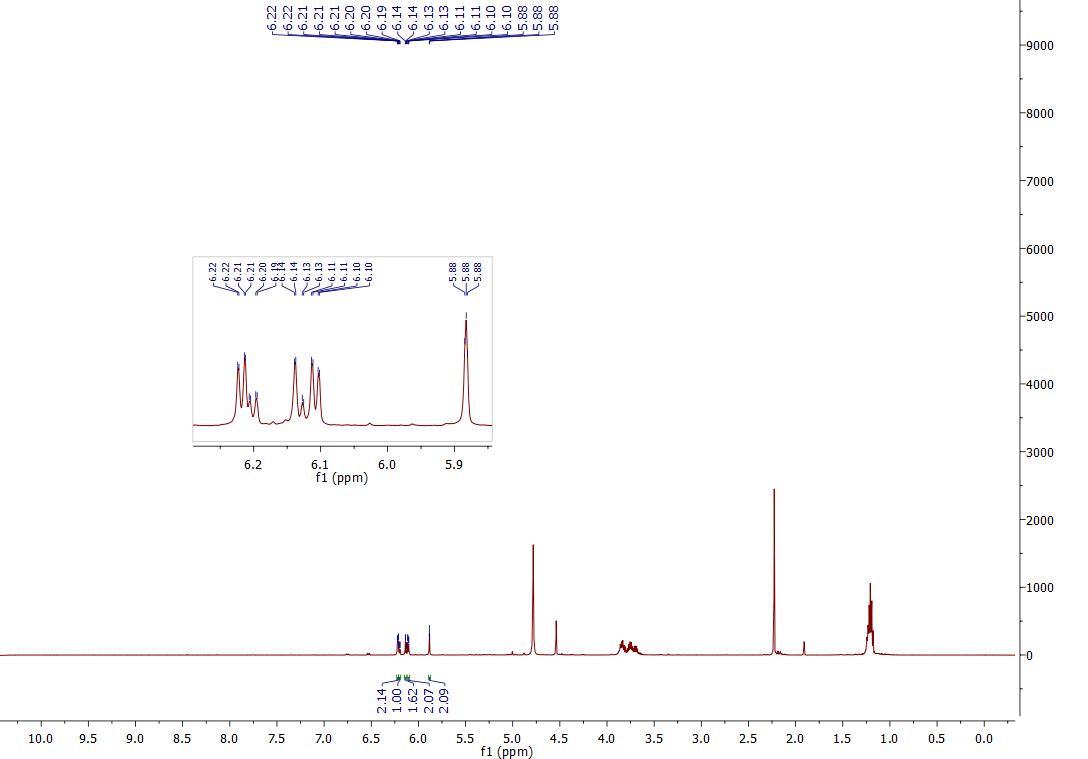
**

**Fig. S5. ^1^H NMR spectrum of DOPE (K_2_DPO_4_/KD_2_PO_4_ in D_2_O)**

**
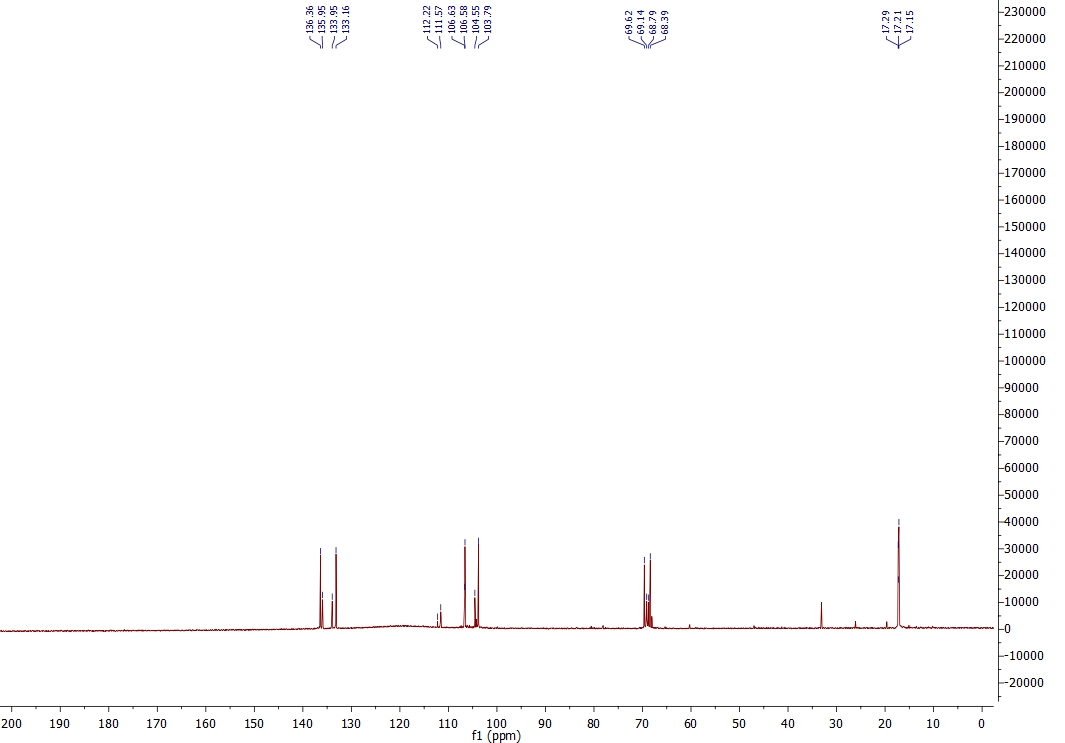
**

**Fig. S6. ^13^C NMR spectrum of DOPE (K_2_DPO_4_/KD_2_PO_4_ in D_2_O)**

**
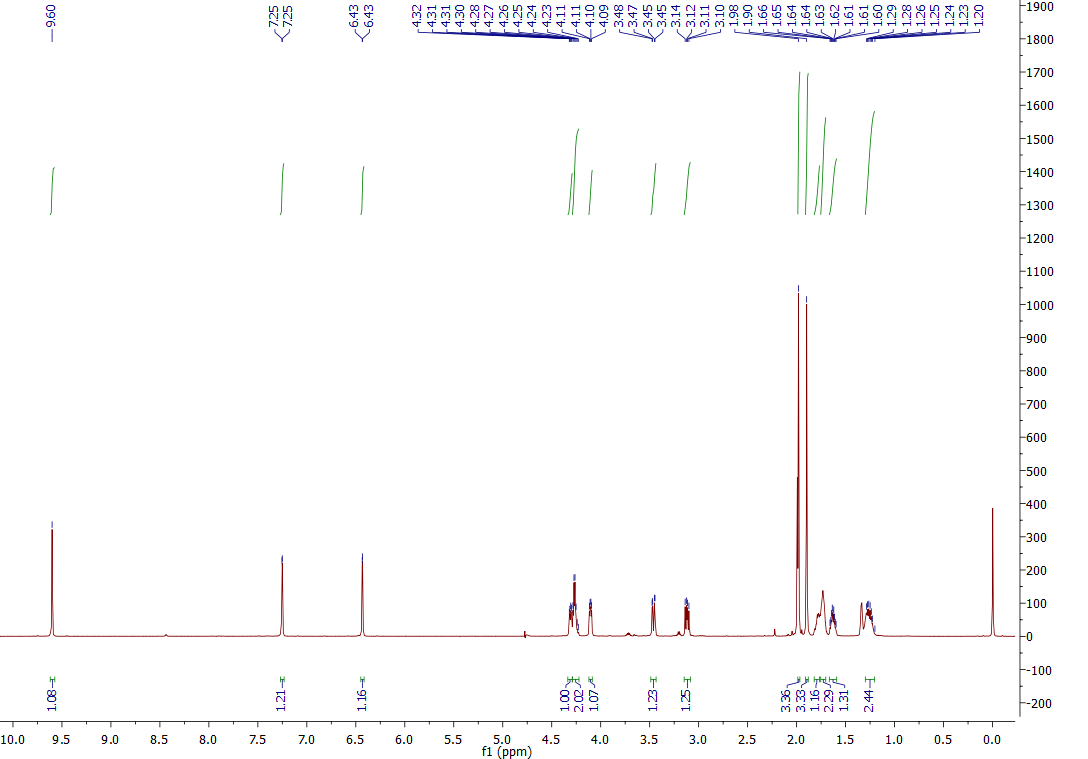
**

**Fig. S7. ^1^H NMR spectrum of product 1 (D_2_O)**


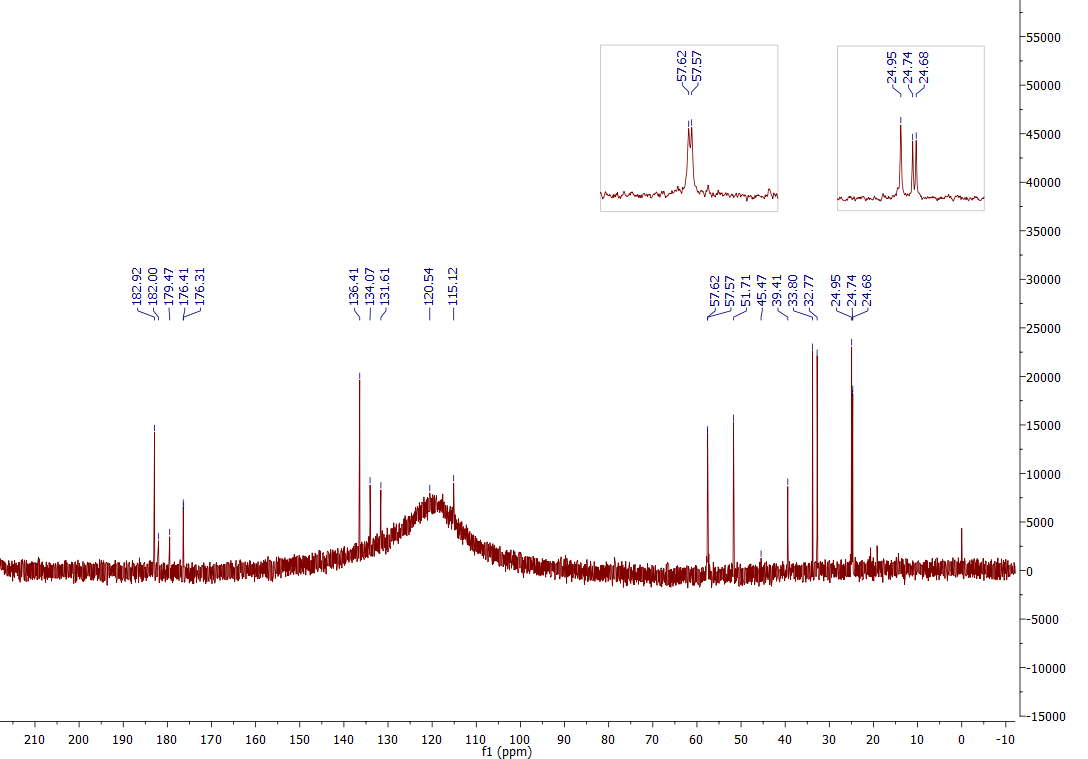


**Fig. S8. ^13^C NMR spectrum of product 1 (D_2_O)**


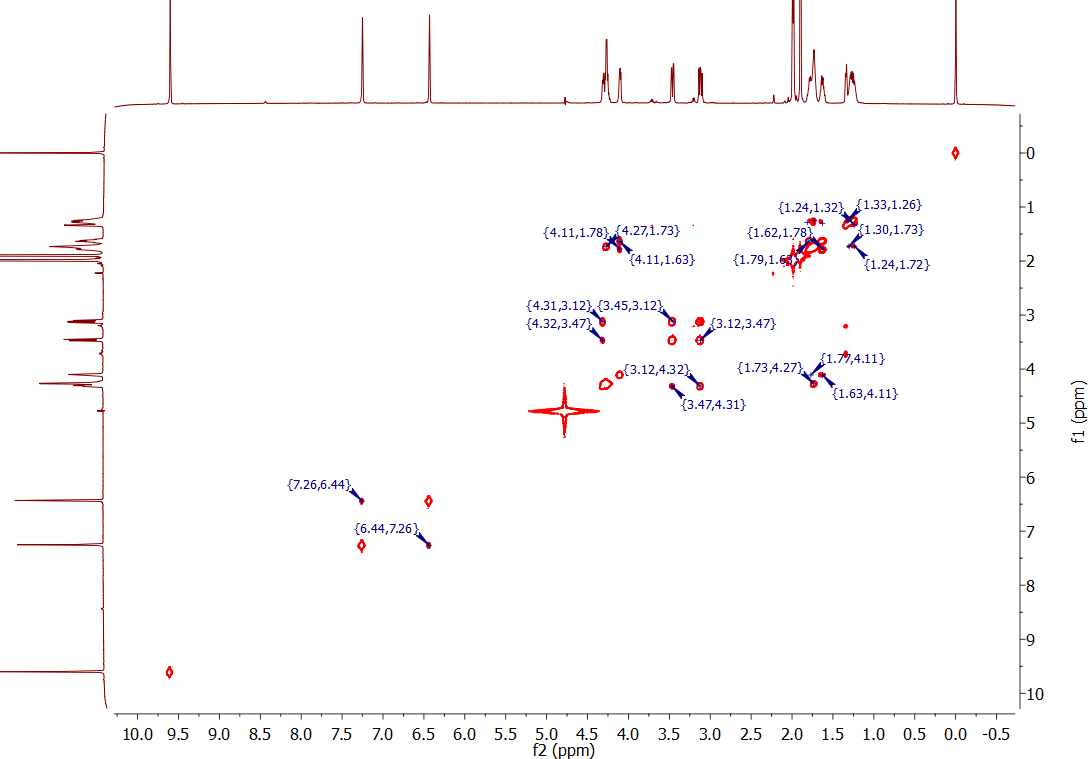


**Fig. S9. COSY spectrum of product 1 (D_2_O)**


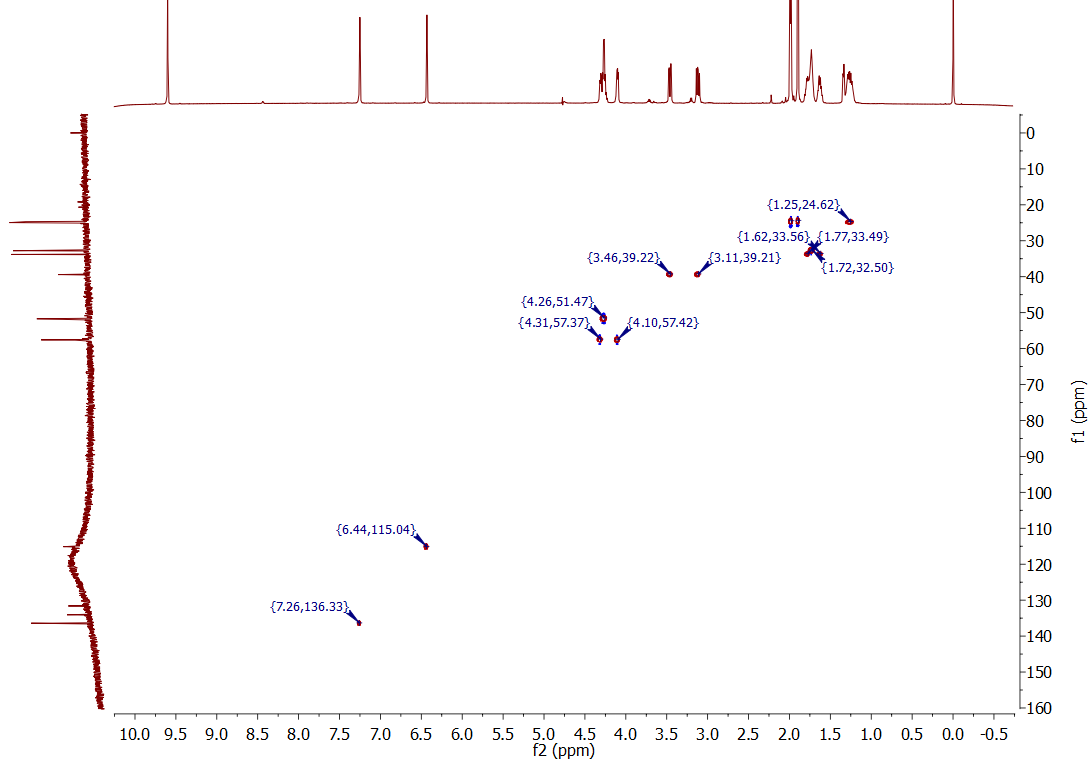


**Fig. S10. HSQC spectrum of product 1 (D_2_O)**


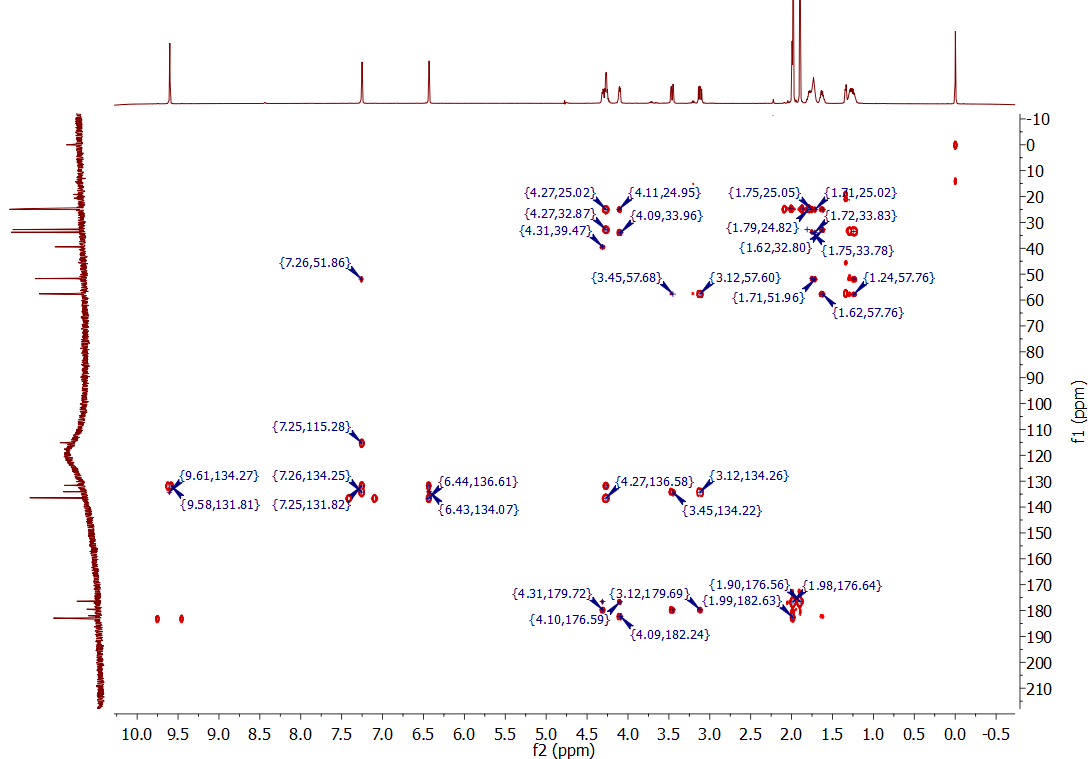


**Fig. S11. HMBC spectrum of product 1 (D_2_O)**


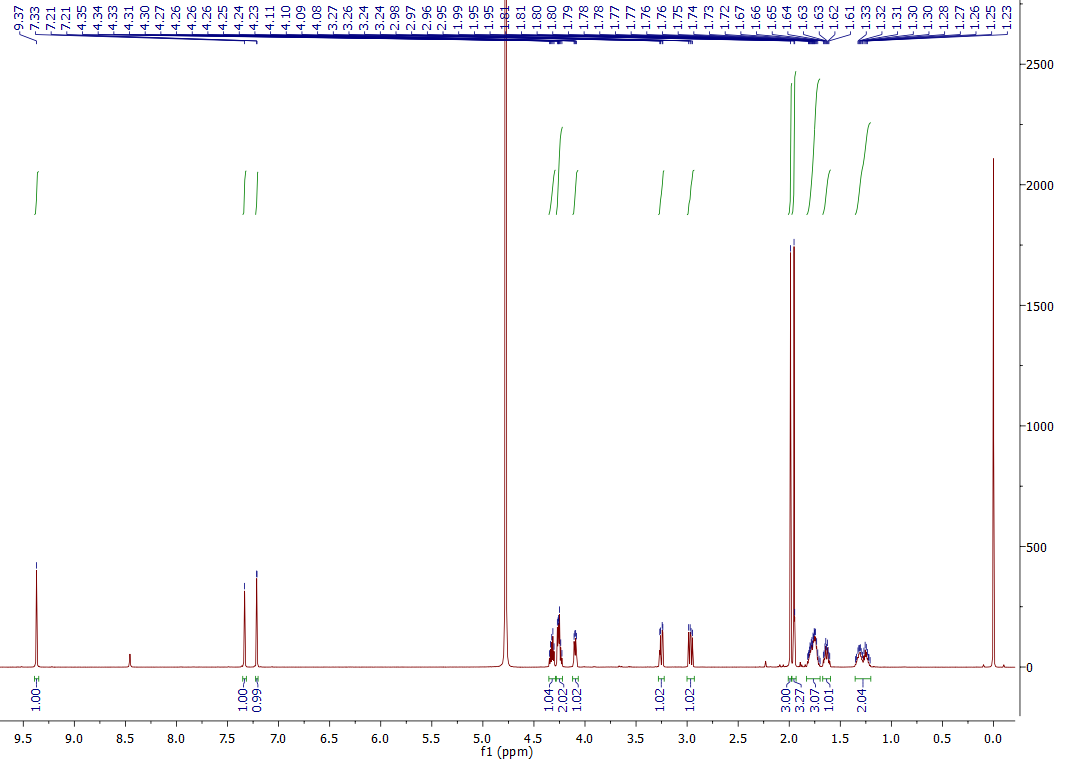


**Fig. S12. ^1^H NMR spectrum of product 2 (D_2_O)**


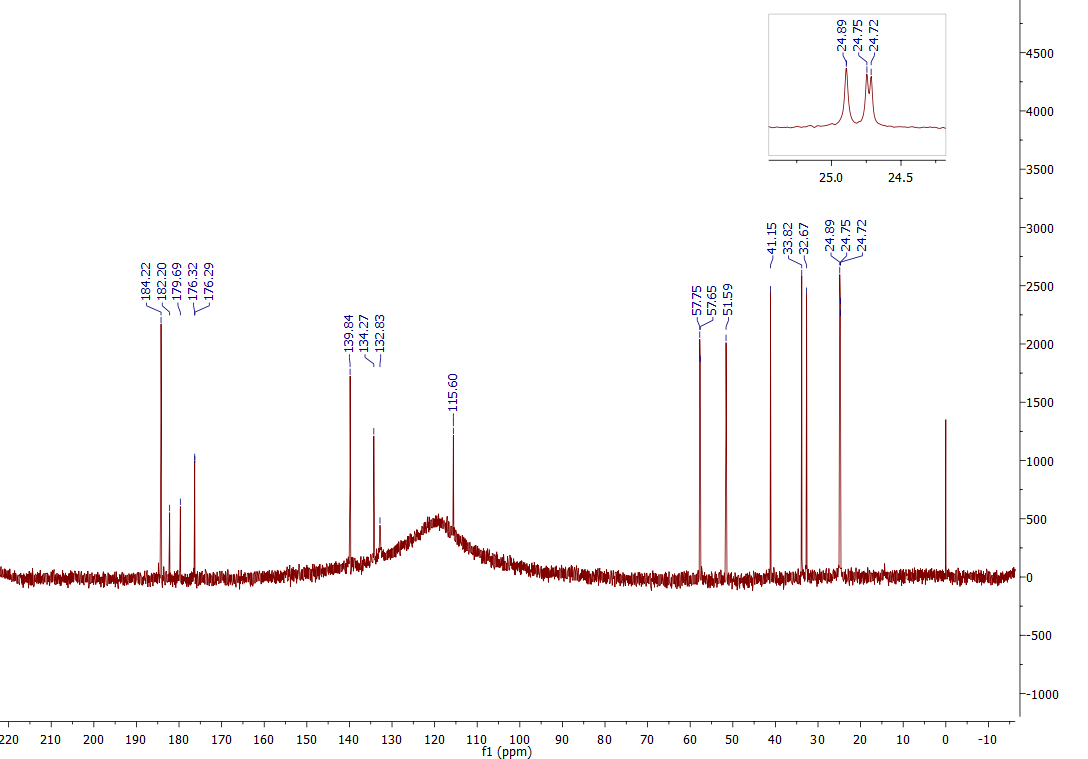


**Fig. S13. ^13^C NMR spectrum of product 2 (D_2_O)**


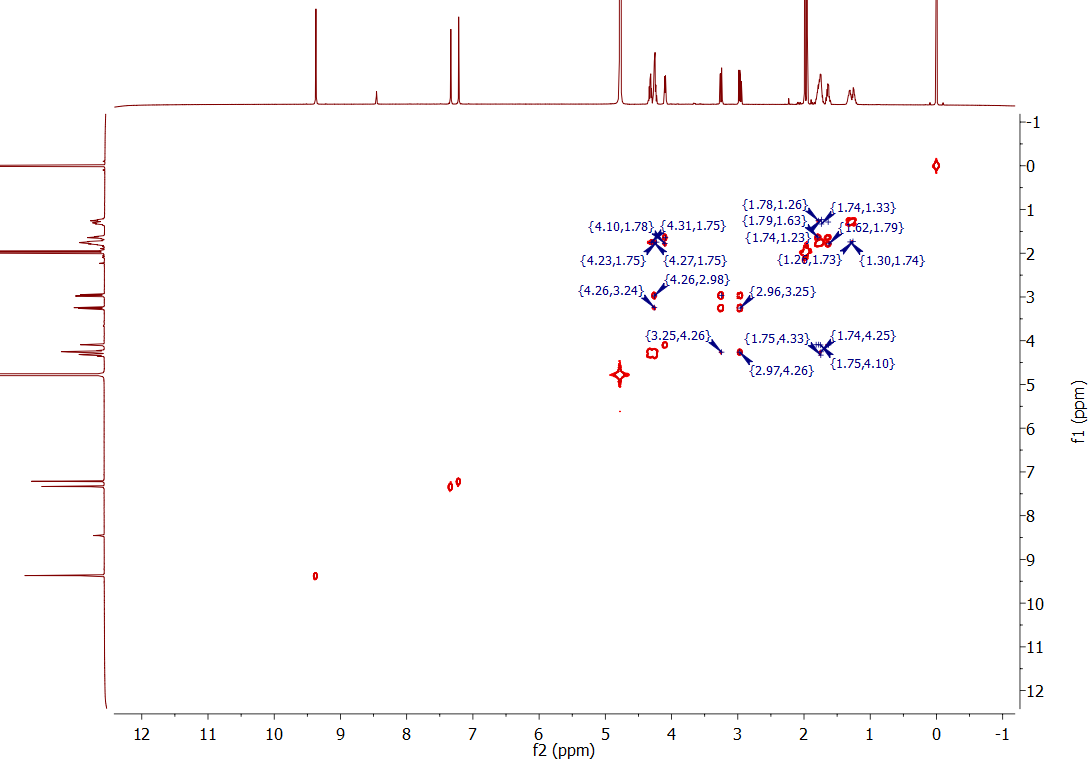


**Fig. S14. COSY spectrum of product 2 (D_2_O)**


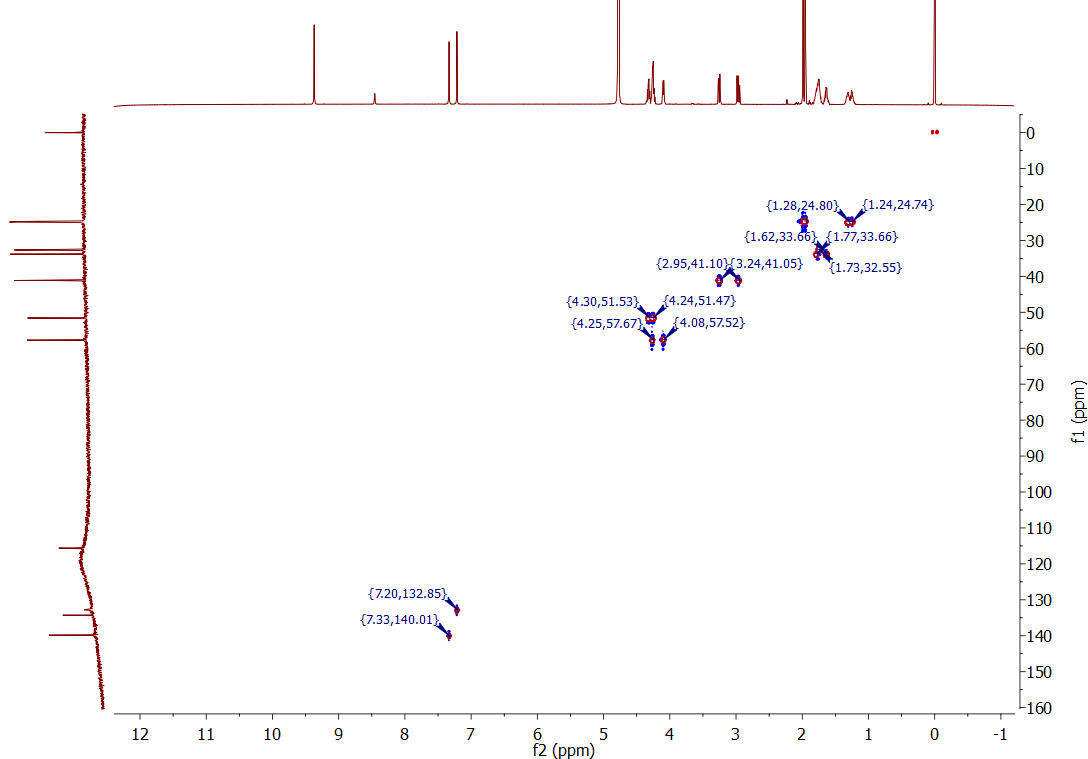


**Fig. S15. HSQC spectrum of product 2 (D_2_O)**


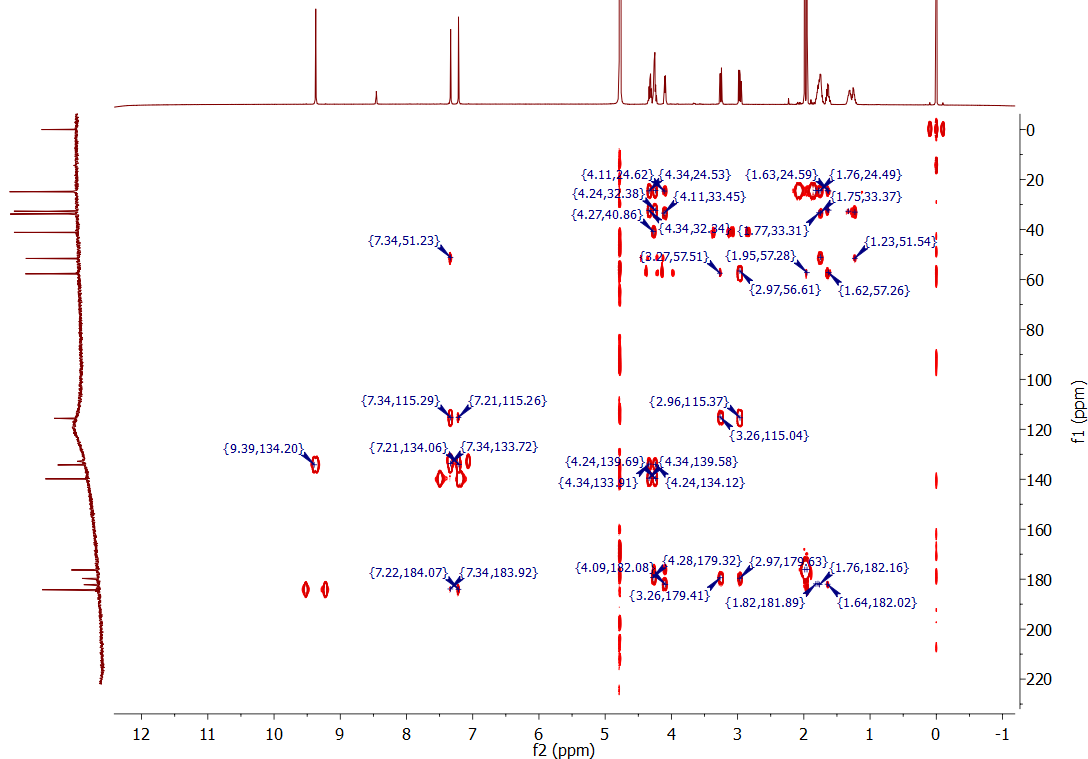


**Fig. S16. HMBC spectrum of product 2 (D_2_O)**


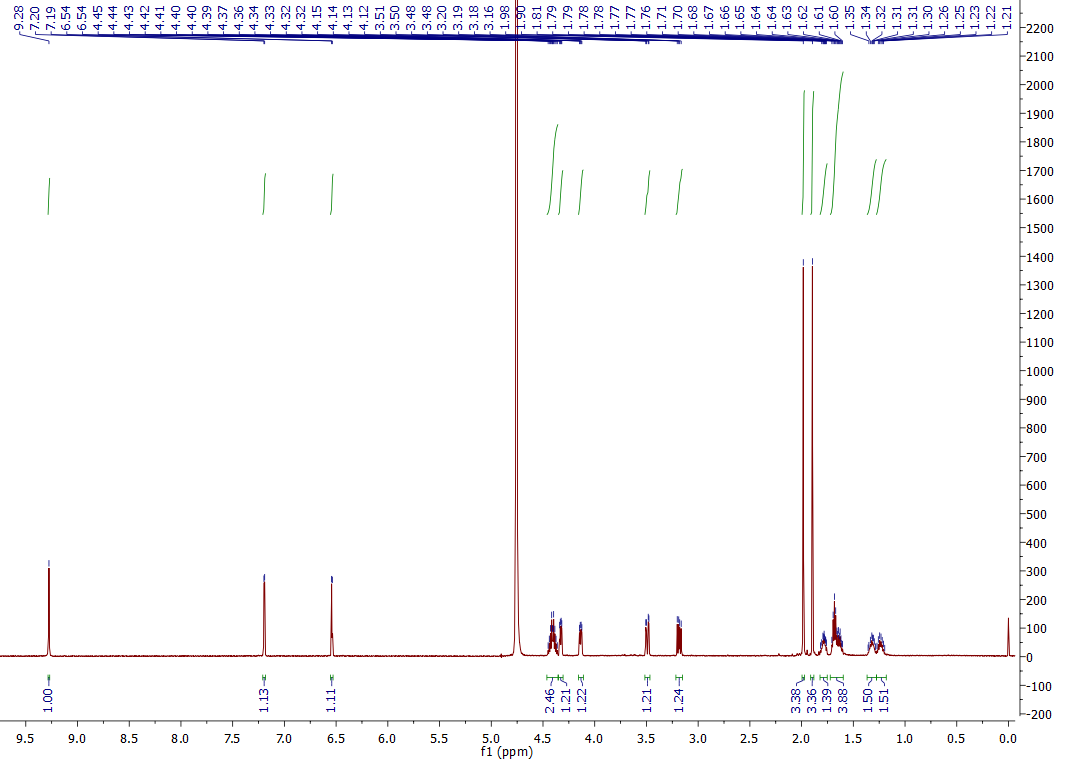


**Fig. S17. ^1^H NMR spectrum of product 3 (D_2_O)**


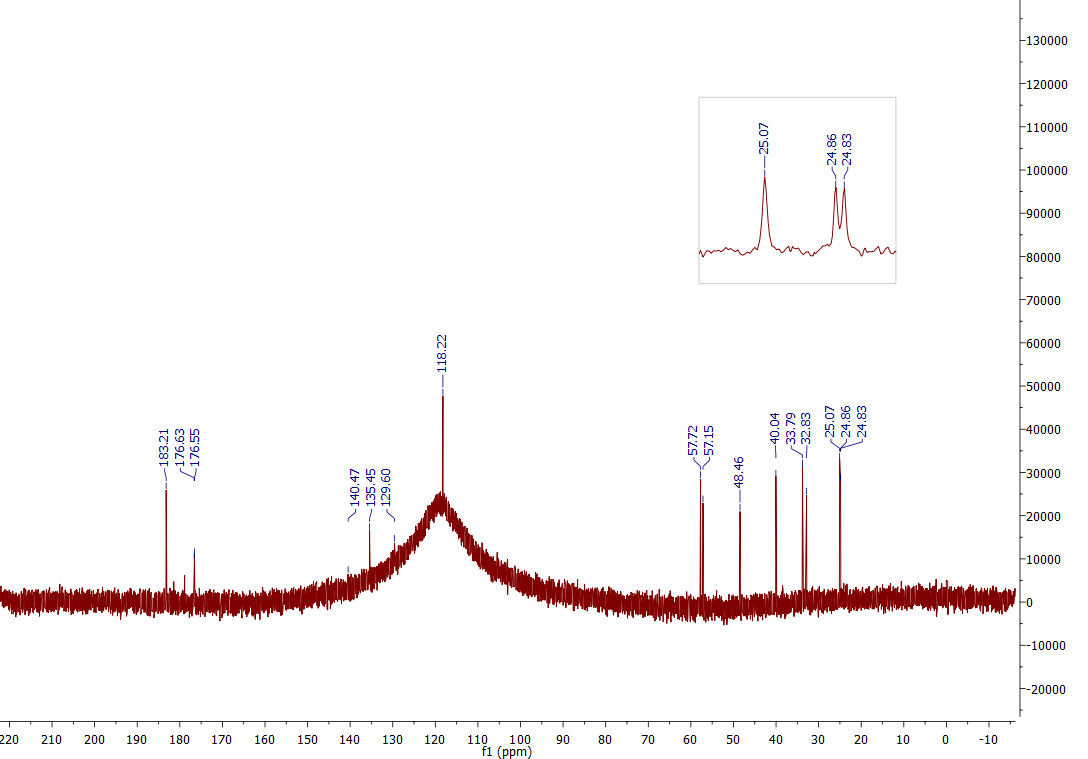


**Fig. S18. ^13^C NMR spectrum of product 3 (D_2_O)**


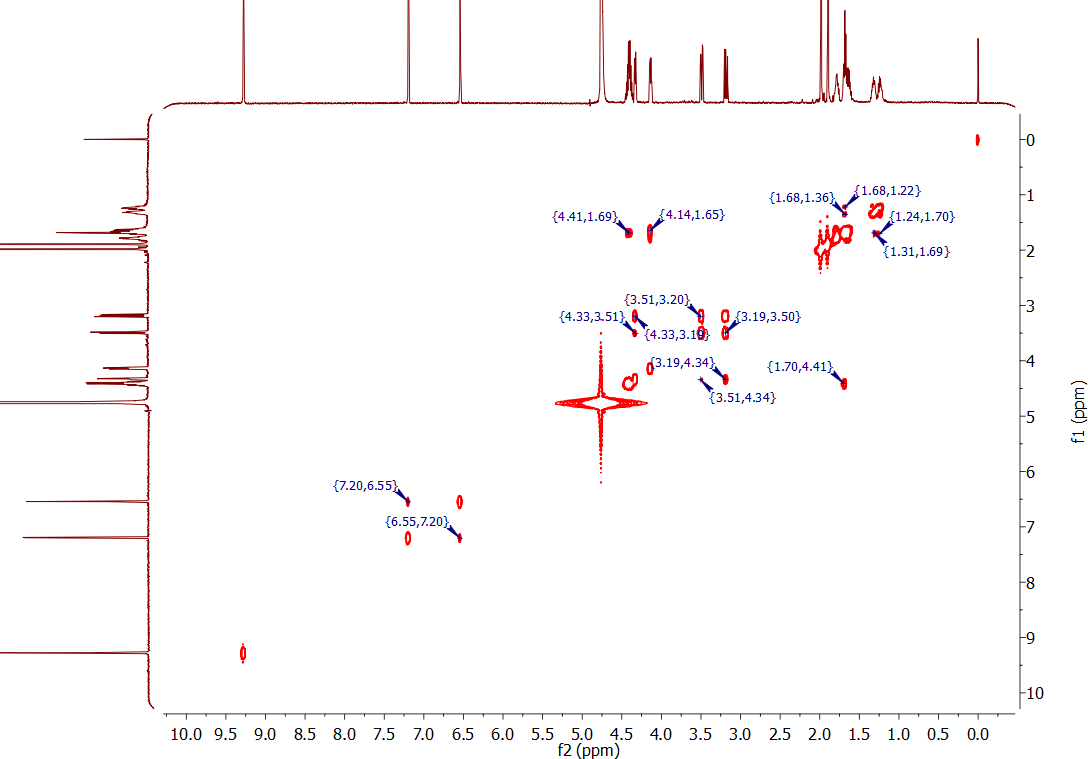


**Fig. S19. COSY spectrum of product 3 (D_2_O)**


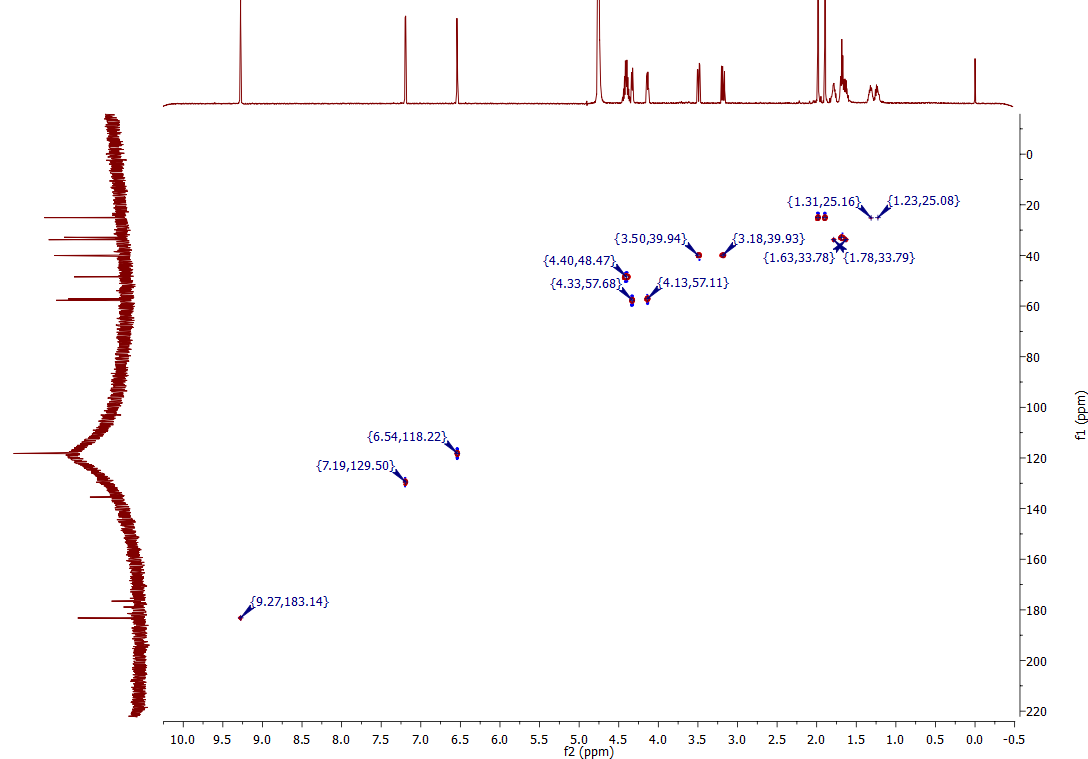


**Fig. S20. HSQC spectrum of product 3 (D_2_O)**


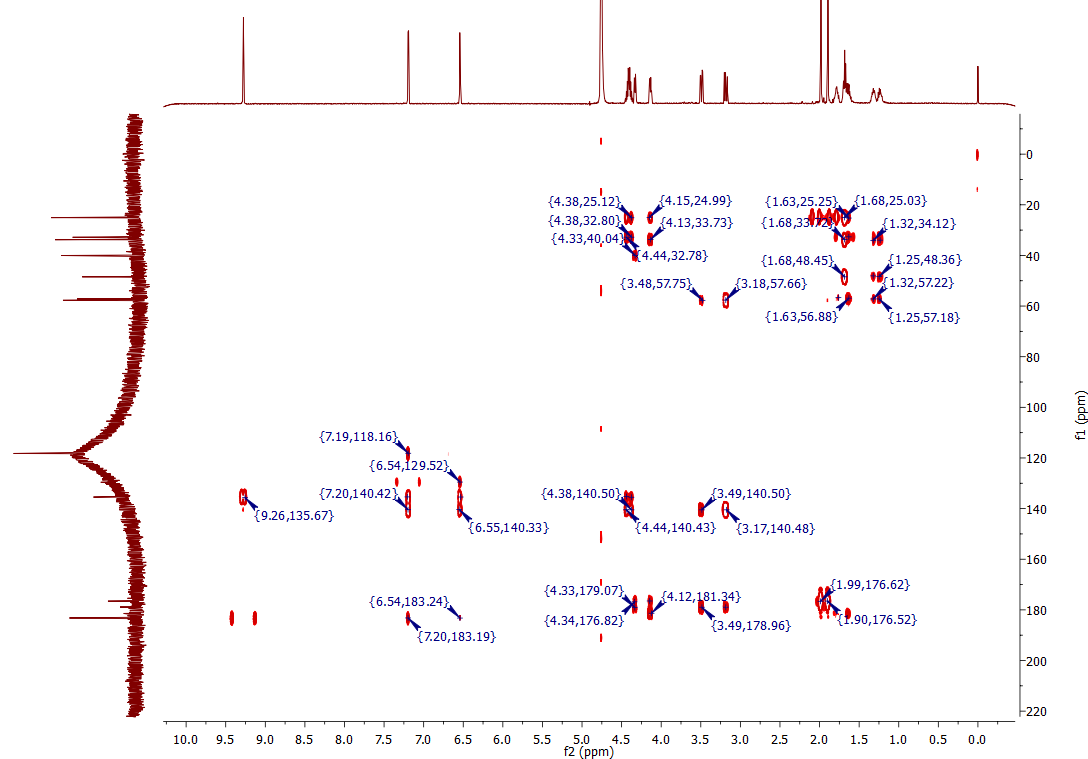


**Fig. S21. HMBC spectrum of product 3 (D_2_O)**

**Preparation of 5,5-diethoxy-4-oxopent-2-enal (DOPE)**

5,5-Diethoxy-4-oxopent-2-enal was obtained by treatment of 2-furaldehyde diethyl acetal (FDA) with 1.5 equivalent of dimethyldioxirane-*d*_6_ (DMDO) in acetone at room temperature. The oxidation of FDA by DMDO was followed by ^1^H NMR spectroscopy and was found to be completed after the reaction time of 2 h. The formation of DOPE was indicated by the appearance of signals of one aldehyde proton at 10.14 ppm (H-C1) and an olefinic protons at 7.35 ppm (H-C2) and 6.36 ppm (H-C3). A signal at 7.35 ppm had coupling constants of 11.99 Hz and 7.08 Hz, identical to the coupling constants of the doublets at 10.14 ppm and 6.36 ppm, respectively (Fig.S22 and Fig.S4). The obtained oxoaldehyde was stable at room temperature. The solution containing this compound was concentrated in a stream of argon and used without further purification.

**Fig. S22. Major chemical shifts (δ) and coupling constant values observed in the ^1^H NMR spectrum (acetone-*d*_6_) of DOPE.**

**Hydration of DOPE**

DOPE was dissolved in 0.1M K_2_DPO_4_/KD_2_PO_4_ (pH^*^ = 7.4) and subjected to NMR studies performed at 37 ˚C. In the ^1^H NMR spectrum no aldehyde proton signal was observed. Apart from the signals derived from the CH(OCH_2_CH_3_) functionality, signals at δ = 5.88, 6.11, 6.14, 6.20 and 6.22 ppm were seen (Fig. S23).


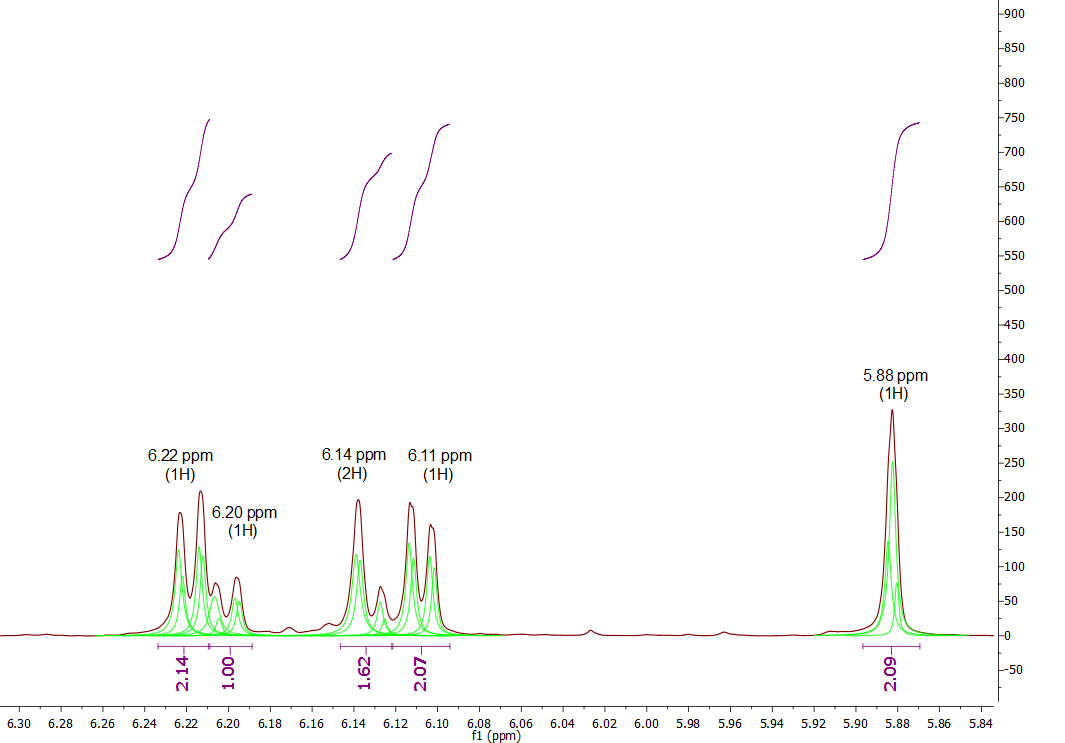


**Fig. S23. Fragment of the ^1^H NMR spectrum (D_2_O) of DOPE hydrates showing vinylic and methine protons signals.**

According to the literature data [1], signals appeared in the range of δ = 6.11-6.22 ppm are characteristic for vinylic protons in cyclic hydrates. The two dd signals (δ = 6.22 and 6.11 ppm) having an integral intensity equal to 2 and ^3^*J*_vic_ = 5.91 Hz, were assigned to vinylic protons of one from two possible isomeric hydrates. The dd signal appeared at δ = 6.20 ppm (*J* = 5.88 Hz) having an integral intensity equal to 1 was assigned to olefinic proton of the second isomer. The multiplet observed in the ^1^H NMR spectrum at δ = 6.14 ppm, correlated in the HSQC spectrum (Fig. S24) with the carbon atom signals appeared at δ = 135.95 and 104.64 ppm. This suggested that the multiplet contains signals derived from both vinylic (δ = 6.14 ppm) and methine (δ = 6.15 ppm) protons. The singlet signal observed at δ = 5.88 ppm was assigned to the methine proton of the second isomeric hydrate. On the basis of the literature data [2,3], the signal was assigned to the proton located in the *trans* position toward OH group (Fig. S25). The signal at δ = 6.15 ppm was assigned to a proton in the *cis* position relative to the C2-OH group (Fig. S25). Based on the information obtained from the ^1^H, ^13^C and HSQC NMR spectra (Fig S5, S6 and S24) and the literature data [3,4], the vinyl proton signals were assigned to the corresponding configurational isomers (Fig. S25).


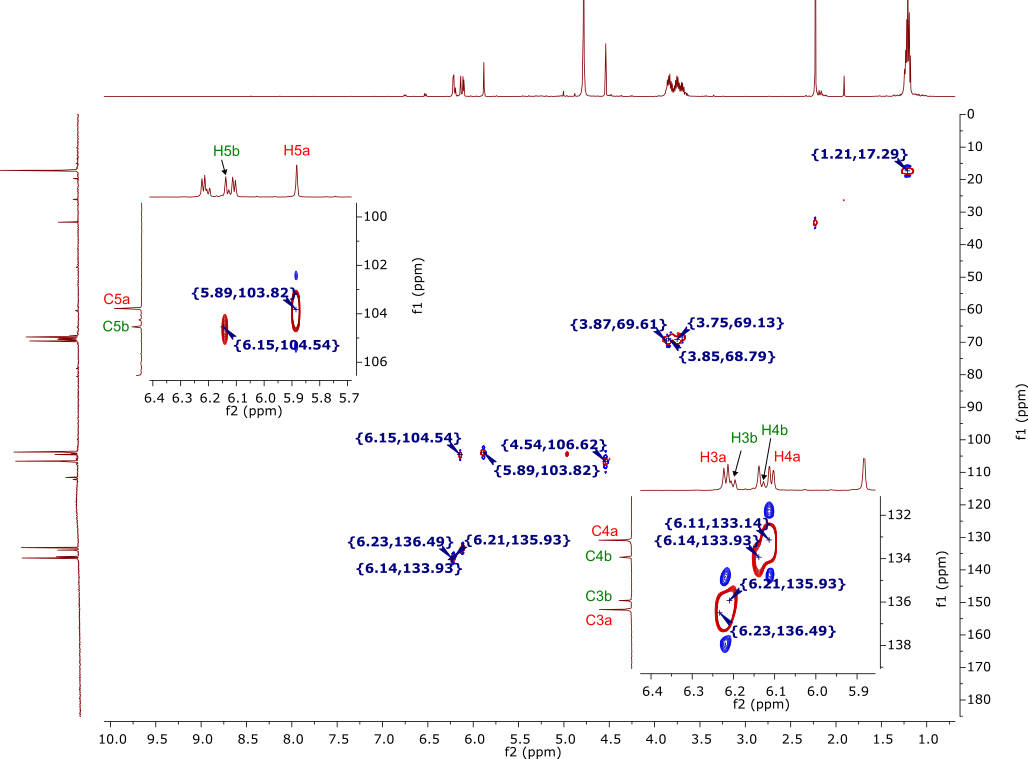


**Fig. S24. HSQC spectrum (D_2_O) of DOPE hydrates**

**Fig. S25. Chemical shift values observed for vinylic and methine protons in the ^1^H NMR spectrum (D_2_O) of the isomeric DOPE hydrates and their corresponding carbons chemical shifts obtained from the HSQC spectrum.**

Ratio of integration of methine and vinylic protons in the *cis* and *trans* isomers was approximately 2.5:1, indicating higher amount of the *cis* one. DOPE molecule contains two carbonyl groups which differ in the reactivity towards nucleophilic agents. It is generally known that aldehydes are more reactive and show higher tendency to form hydrates. Therefore the mechanism of the DOPE hydrates formation assumes nucleophilic addition of water molecule to the aldehyde group followed by intramolecular reaction between the keto group and the C1-OH hydroxyl group yielding the cyclic hydrates. Due to the fact that the attack of the OH group can occur from both *Re* and *Si* sides, mixture of two diastereomeric products is formed.

**UV spectra of the studied compounds**

**Fig. S26. UV-Vis spectra of Product 1 (A) and Product 2 (B). The spectra were recorded with a diode array detector as the compounds eluted from the HPLC column. (For analysis conditions see the Experimental section in the manuscript).**

**Fig. S27. UV-Vis spectrum of Product 3. The spectrum was recorded with a diode array detector as the compound eluted from the HPLC column. (For analysis conditions see the Experimental section in the manuscript).**

**Mass spectra of the studied compounds**


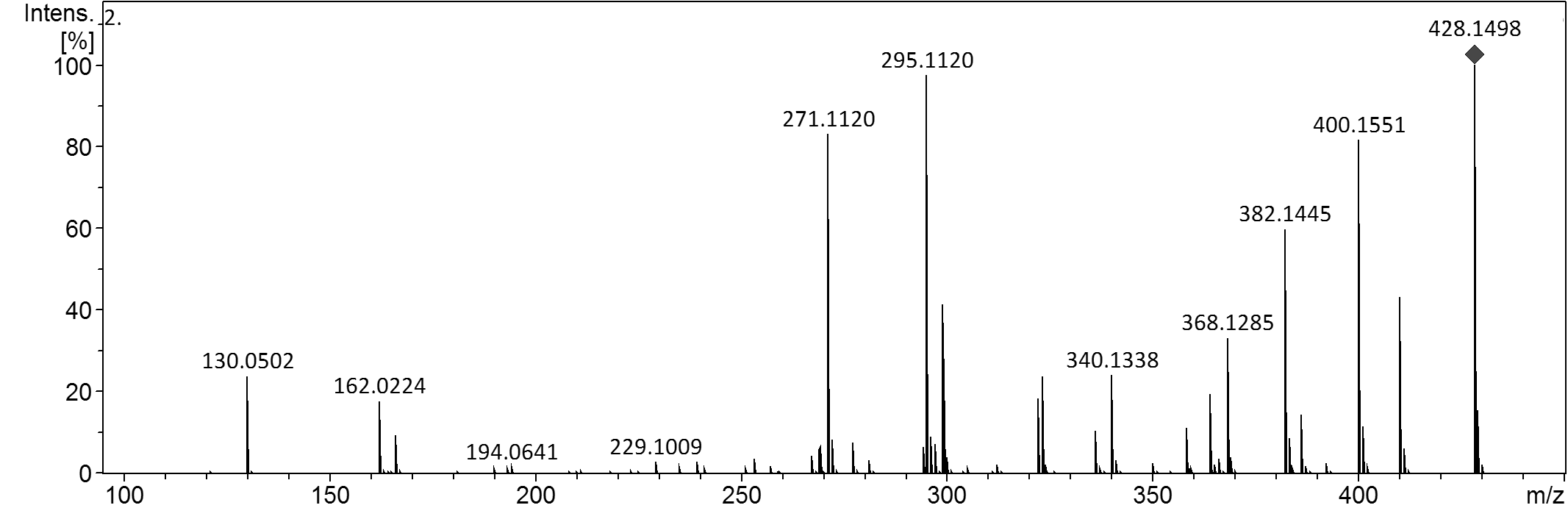


**Fig.S28.** **Positive ions spectrum of product 1**


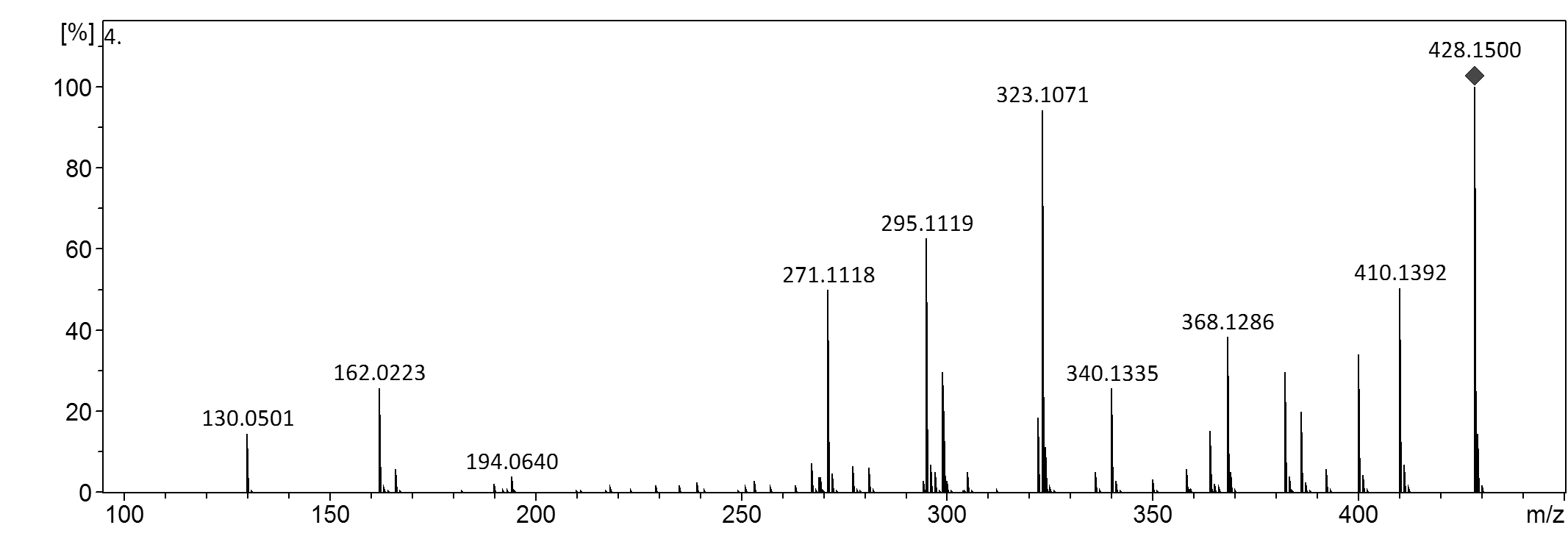


**Fig.S29.** **Positive ions spectrum of product 2**


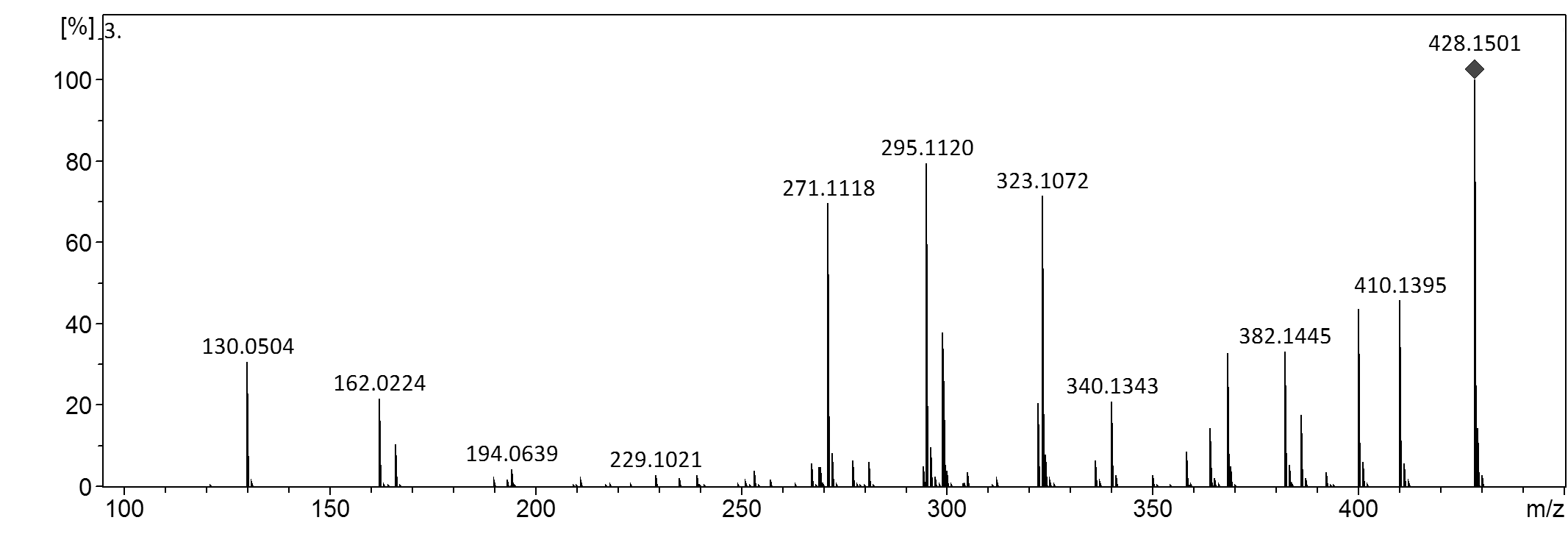


**Fig.S30.** **Positive ions spectrum of product 3**

[1] L.-J. Chen, S.S. Hecht, L.A. Peterson, Identification of cis-2-Butene-1,4-dial as a Microsomal Metabolite of Furan, Chem. Res. Toxicol. 8 (1995) 903–906. https://doi.org/10.1021/tx00049a001.

[2] K. Yoshida, T. Fueno, Concurrent Anodic Cyanation and Methoxylation of Methylated Furans. Oxidation Potential and Reactivity, and Stereochemical Control of Addition, Bull. Chem. Soc. Jpn. 60 (1987) 229–240. https://doi.org/10.1246/bcsj.60.229.

[3] O.S. Tee, B.E. Swedlund, On the reaction of furan with bromine in aqueous solution. Observation of the slow hydration of malealdehyde, Can. J. Chem. (2011). https://doi.org/10.1139/v83-378.

[4] H. Greuter, T. Winkler, Die Struktur der durch Halogenierung von Furfural in Wasser gebildeten Oxydationsprodukte, Helv. Chim. Acta (1978) 61(8), 3103-7. https://doi.org/10.1002/hlca.19780610838.
